# Asthma and genes encoding components of the vitamin D pathway

**DOI:** 10.1186/1465-9921-10-98

**Published:** 2009-10-24

**Authors:** Yohan Bossé, Mathieu Lemire, Audrey H Poon, Denise Daley, Jian-Qing He, Andrew Sandford, John H White, Alan L James, Arthur William Musk, Lyle J Palmer, Benjamin A Raby, Scott T Weiss, Anita L Kozyrskyj, Allan Becker, Thomas J Hudson, Catherine Laprise

**Affiliations:** 1 Institut universitaire de cardiologie et de pneumologie de Québec, Québec, Canada; 2 Laval University Hospital Research Center (CRCHUL), Québec, Canada; 3 Ontario Institute for Cancer Research, Toronto, Canada; 4 The Channing Laboratory, Department of Medicine, Brigham and Women's Hospital, Boston, MA, USA; 5 Harvard Medical School, Boston, MA, USA; 6 James Hogg iCAPTURE Centre for Cardiovascular and Pulmonary Research, St Paul's Hospital, University of British Columbia, Vancouver, Canada; 7 Departments of Physiology and Medicine, McGill University, Montreal, Canada; 8 West Australian Sleep Disorders Research Institute, Sir Charles Gairdner Hospital, Western Australia; 9 Department of Respiratory Medicine, Sir Charles Gairdner Hospital, Western Australia; 10 UWA Centre for Genetic Epidemiology and Biostatistics, The University of Western Australia, Western Australia; 11 Division of Pulmonary and Critical Care Medicine, Department of Medicine, Brigham and Women's Hospital, Boston, MA, USA; 12 The Center for Genomics Medicine, Department of Medicine, Brigham and Women's Hospital, Boston, MA, USA; 13 Department of Pediatrics and Child Health, Faculty of Medicine, University of Manitoba, Winnipeg, Canada; 14 Université du Québec à Chicoutimi, Chicoutimi, Canada; 15 Community Genomic Medicine Centre, University of Montreal, Chicoutimi University Hospital, Chicoutimi, Canada

## Abstract

**Background:**

Genetic variants at the vitamin D receptor (VDR) locus are associated with asthma and atopy. We hypothesized that polymorphisms in other genes of the vitamin D pathway are associated with asthma or atopy.

**Methods:**

Eleven candidate genes were chosen for this study, five of which code for proteins in the vitamin D metabolism pathway (*CYP27A1*, *CYP27B1*, *CYP2R1*, *CYP24A1*, *GC*) and six that are known to be transcriptionally regulated by vitamin D (*IL10*, *IL1RL1*, *CD28*, *CD86*, *IL8*, *SKIIP*). For each gene, we selected a maximally informative set of common SNPs (tagSNPs) using the European-derived (CEU) HapMap dataset. A total of 87 SNPs were genotyped in a French-Canadian family sample ascertained through asthmatic probands (388 nuclear families, 1064 individuals) and evaluated using the Family Based Association Test (FBAT) program. We then sought to replicate the positive findings in four independent samples: two from Western Canada, one from Australia and one from the USA (CAMP).

**Results:**

A number of SNPs in the *IL10*, *CYP24A1*, *CYP2R1*, *IL1RL1 *and *CD86 *genes were modestly associated with asthma and atopy (p < 0.05). Two-gene models testing for both main effects and the interaction were then performed using conditional logistic regression. Two-gene models implicating functional variants in the *IL10 *and *VDR *genes as well as in the *IL10 *and *IL1RL1 *genes were associated with asthma (p < 0.0002). In the replicate samples, SNPs in the *IL10 *and *CYP24A1 *genes were again modestly associated with asthma and atopy (p < 0.05). However, the SNPs or the orientation of the risk alleles were different between populations. A two-gene model involving *IL10 *and *VDR *was replicated in CAMP, but not in the other populations.

**Conclusion:**

A number of genes involved in the vitamin D pathway demonstrate modest levels of association with asthma and atopy. Multilocus models testing genes in the same pathway are potentially more effective to evaluate the risk of asthma, but the effects are not uniform across populations.

## Background

Asthma is a heterogeneous respiratory disease characterized by chronic inflammation of the airways associated with recurrent symptoms that range from mild to debilitating [1]. Asthma is in large part attributable to genetic factors [2]. However, identifying the causal genes has been a daunting task due to the inherent complexity of the disease as well as methodological issues related to finding genes of complex diseases [3]. The emerging picture from the literature suggests hundreds of genes are associated with asthma or asthma-related phenotypes [4,5]. Major efforts are currently underway to validate these genes in larger populations as well as to identify novel genes using new technology-driven approaches such as genome-wide single-nucleotide-polymorphism (SNP) association studies [6-8].

The innate and adaptive immune systems play an important role in the pathogenesis of asthma. Many genes involved in inflammation and immunoregulation pathways have been associated with asthma [3]. The immune system is complex in nature with multiple redundant and interfering pathways. Recently, the vitamin D pathway has emerged as a new pathway contributing to the outcome of immune responses [9-12]. The vitamin D pathway has long been recognized for its endocrine actions on bone and mineral homeostasis. However, growing knowledge has elucidated autocrine and paracrine roles for the vitamin D system with respect to cell growth, proliferation and differentiation as well as in immune regulation [13]. The biologically active form of vitamin D (1α,25-dihydroxyvitamin D_3_), also known as calcitriol, mediates its effect by binding to the nuclear vitamin D receptor (VDR). Upon activation, the VDR ligand/receptor complex alters the transcription rate of many genes involved in a wide spectrum of biological responses [14].

The hypothesis that the vitamin D pathway plays a role in autoimmune diseases such as asthma, originates from the identification of VDR in immunological relevant cells, including antigen-presenting cells and activated T lymphocytes [15,16]. How VDR affects immune cell populations, cytokine secretion, and production is not entirely known, but previous evidence suggests that VDR activation may cause a developmental shift of T helper (Th) cells toward type 2 [17,18]. The hypothesis that VDR plays a role in asthma was also reinforced by the resistance of VDR knock-out mice to experimentally induced asthma [19]. These mice fail to develop airway inflammation, eosinophilia, or airway hyperresponsiveness, despite high IgE concentration and elevated Th2 cytokines. Recently, a functional polymorphism (FokI, rs2228570) in the *VDR *gene was shown to have a functional impact on the immune system by interfering with the signaling of transcription factors important in immune-mediated diseases such as NF-κB and NFAT [20]. Taken together, these studies clearly support *VDR *as a possible candidate gene for asthma.

Two groups co-reported that genetic variants within the *VDR *gene were associated with asthma [21,22]. In a French-Canadian founder population, Poon et al. [21] demonstrated that six SNPs located between intron 2 and exon 9 spanning 28 kb of the *VDR *gene were associated with asthma. Linkage disequilibrium (LD) patterns within this population revealed the presence of two blocks (block 1 and 2) containing 3 and 4 common haplotypes, respectively. One haplotype within each block was overtransmitted to affected offspring. By sequencing the promoter, exons and surrounding regions, they excluded novel missense mutations that could explain the observed association. In a second study, Raby et al. [22] found significant associations between *VDR *variants and asthma in two independent studies. They first screened seven candidate genes that map to the centromeric region of chromosome 12 in the Childhood Asthma Management Program (CAMP) study. Only one SNP located in the *VDR *gene demonstrated evidence of association with asthma. Consistent with the French-Canadian population, two LD blocks were observed, each with three common haplotypes. The 3' haplotype block in the CAMP study was significantly associated with asthma. To exclude the possibility that neighboring genes cause the association, the authors genotyped 29 SNPs in a 330 kb region surrounding the *VDR *gene. None of these SNPs were associated with asthma, leaving *VDR *as the most likely causal gene. Their finding was then replicated in the Nurses' Health Study (NHS) [22]. In that study, four of the six genotyped SNPs within the *VDR *gene were associated with asthma. However, it should be noted that the direction of the association in NHS was opposite to the effects seen in CAMP, but similar to the findings in the French-Canadian population. Taken together, these data suggested that the *VDR *locus harbors variants that contribute to asthma, but the orientation of the risk allele is inconsistent across populations.

Numerous metabolic pathways are likely to play a major role in complex diseases. It is necessary to study the components of these pathways to gain a more comprehensive genetic view of the susceptibility conferred by variants located in closely related genes [23,24]. Accordingly, we hypothesized that polymorphisms in other genes involved in the vitamin D system are associated with asthma or atopy.

## Methods

### Population

Subjects were from the Saguenay^_^Lac-Saint-Jean (SLSJ) asthma study, which consists of French-Canadian families ascertained through asthmatic probands. Probands were included in the study if they fulfilled at least two of the following criteria: 1) a minimum of three clinic visits for acute asthma within one year; 2) two or more asthma-related hospital admissions within one year; or 3) steroid dependency, defined by either six month's use of oral, or one year's use of inhaled corticosteroids. A total of 1064 individuals from 388 nuclear families were included in the present analyses. Families were included in the study if at least one parent was available for phenotypic assessment, at least one parent was unaffected, and all four grandparents were of French-Canadian origin. Family members were considered asthmatics if both a self-reported history of asthma and a history of physician-diagnosed asthma were recorded, or by clinical evaluation following a methacholine provocation test. Skin-prick tests were performed for 26 inhalant allergens and subjects were considered atopic if they had at least one positive response (wheal diameter ≥ 3 mm at 10 min) [25]. Spirometry, methacholine challenge and IgE measurements are described in detail elsewhere [21]. Table 1 presents the characteristics of the subjects. The SLSJ local ethics committee approved the study, and all subjects gave informed consent.

**Table 1:** Characteristics of the subjects in the Saguenay^_^Lac-Saint-Jean study.

	All subjects (n = 1064)	Probands (n = 210)	Affected members (n = 320)	Unaffected members (n = 534)
Age (years)	39.7 ± 22.1	17.6 ± 9.4	40.0 ± 19.5	48.3 ± 21.1
Male: Female ratio	0.80	0.86	0.68	0.85
Mean age of onset (years)	16.5 ± 17.0	7.4 ± 7.6	22.3 ± 18.7	NA
FEV_1 _(% predicted)	94.1 ± 19.8	92.5 ± 16.1	88.9 ± 23.2	99.1 ± 17.2
PEFR (%)	6.4 ± 4.6	7.3 ± 4.2	7.4 ± 5.9	5.0 ± 2.9
PC_20 _(mg/ml)	21.1 ± 24.9	5.1 ± 8.5	8.8 ± 15.4	39.4 ± 24.9
Serum IgE (mg/L)	452.7 ± 1619.2	720.3 ± 1919.9	531.4 ± 1960.8	244.8 ± 1005.9
Atopy (n)	593 (56.3%)	171 (81.8%)	218 (69.2%)	204 (38.6%)
Smoking Status (n)				
Never	551 (52.6%)	173 (84.0%)	154 (48.7%)	224 (42.7%)
Ex-smoker	291 (27.8%)	11 (5.3%)	100 (31.7%)	180 (34.3%)
Smoker	205 (19.6%)	22 (10.7%)	62 (19.6%)	121 (23.1%)

Values are means ± SD for quantitative variables.

FEV_1_, Forced expiratory volume in one second; PC_20_, Concentration of methacholine inducing a 20% fall in FEV_1_; PEFR, Peak expiratory flow rate (morning-evening variation).

### Replication samples

Data from the Canadian Asthma Primary Prevention Study (CAPPS) study, the Study of Asthma Genes and the Environment (SAGE) birth cohort and the Busselton Health Study (BHS) were used to replicate the findings. The Childhood Asthma Management Program (CAMP) study was also used to replicate a specific gene-gene (VDR-IL10) interaction models. The CAPPS and SAGE studies have been described elsewhere [26]. Briefly, the CAPPS study was initiated in 1995 to assess the effectiveness of a multifaceted intervention program in the primary prevention of asthma in high-risk infants [27,28]. High-risk infants were identified before birth as having at least one first-degree relative with asthma or two first-degree relatives with other IgE-mediated allergic diseases. A total of 549 children and their parents forming 545 families were enrolled in the study during the second and third trimester of pregnancy. The children were followed since birth and were assessed by a pediatric allergist for the presence of asthma and allergies. Atopy was defined by skin-prick test. A total of 16 allergens were tested and the diagnosis was positive if at least one wheal ≥ 3 mm than the negative control was observed. Children with 7 year follow-up data and DNA were included in the current study (380 children/families). The SAGE study is a population-based cohort of 16,320 children born in the province of Manitoba, Canada, between January 1, 1995 and December 31, 1995. Parents of these children were first survey by mail in 2002. A subset of children was then invited to join the study at age 8-10 years. This subset included children with parent-declared asthma and children without asthma. A total of 723 families were recruited into the study. All recruited children underwent clinical assessment of asthma by a pediatric allergist. Skin prick testing for 16 allergens was used to define atopy. In the two latter studies (SAGE and CAPPS), children affected with asthma/atopy and their parents were genotyped and analysed in trios. In contrast, the BHS was analysed using a case-control design. This study comprised a series of six cross-sectional health surveys that took place every three years from 1966 to 1981 in all adults and children residing in the Shire of Busselton, Western Australia and a follow-up study of all previous participants (residing within and outside this Shire) in 1994/1995. Busselton is a coastal town in the South West region of Western Australia with a population that is predominantly of European origin. In the present case-control study, all subjects (n = 1395, 751 controls and 644 cases) who attended both the 1981 and the 1994 survey and who had a diagnosis of asthma as well as available DNA were included. Subjects were considered to have asthma if they answered yes to the question "Has your doctor ever told you that you had asthma/bronchial asthma?" in a written questionnaire at either survey. Subjects were considered controls if they answered no at both surveys. Skin prick testing for 12 allergens was used to define atopy. Finally, CAMP is a multicentered North American clinical trial designed to investigate the long-term effects of inhaled anti-inflammatory medications in children with mild to moderate asthma [29,30]. A total of 1625 individual members of 428 non-Hispanic white nuclear families were included in the present analyses. This represents the subset non-Hispanic with CAMP families with available SNP genotype data at both the VDR and IL10 loci. The diagnosis of asthma was based on a methacholine provocation test and one or more of the following criteria for at least 6 months in the year before recruitment: 1) asthma symptoms at least two times per week, 2) at least two uses per week of an inhaled bronchodilator, and 3) daily asthma medication. A local ethics committee approved the protocol independently in each study. Written informed consent was obtained from all study participants.

### Gene selection

**Figure 1:**
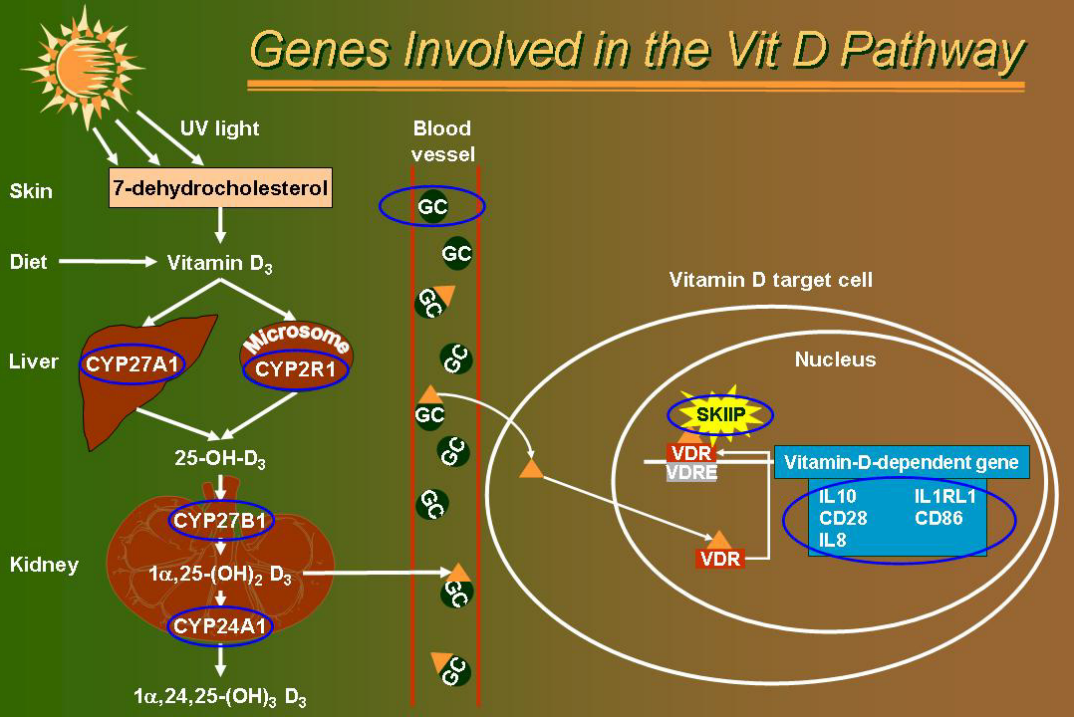
**Genes involved in the vitamin D pathway**. Vitamin D_3 _comes from the diet but is mostly produced in the skin by the photolytic cleavage of 7-dehydrocholesterol. From vitamin D_3_, two enzymatic activation steps are required to produce the biologically active form of vitamin D [1α,25-(OH)_2 _D_3_]. *CYP27A1 *and *CYP2R1 *genes encode enzymes with 25-hydroxylase activity that catalyze the C-25 hydroxylation of vitamin D_3_. A final activation enzyme encoded by *CYP27B1 *subsequently catalyzes the rate-limiting C-1 hydroxylation step in 1α,25-(OH)_2 _D_3 _synthesis. The later enzyme is tightly-regulated in the kidney by calcium homeostatic signals, but also strongly induced by immune inputs (e.g. TLR signaling) in many cells of the immune system [12] (not depicted). The active form of vitamin D, 1α,25-(OH)_2 _D_3 _(orange triangle), is then transport to vitamin D target cells by the vitamin D binding protein (encoded by the *GC *locus) or is metabolically inactivated by the 24-hydroxylase enzyme (encoded by the *CYP24A1 *locus). In vitamin D target cells, 1α,25-(OH)_2 _D_3 _translocates to the nucleus and binds to the vitamin D receptor (VDR). The ligand/receptor complex binds vitamin D response element (VDRE) located in the promoter region of target genes. The DNA-bound complex interacts with nuclear coregulators, such as SKIIP [33], and alters the rate of gene transcription. Five genes having a VDRE or/and being transcriptionally regulated by vitamin D stimulation are shown (blue square). Genes selected for genotyping in the SLSJ study are circled in blue.

Eleven candidate genes were chosen for this study. Figure 1 is a cartoon of the vitamin D pathway that illustrates the implication of each gene selected. Briefly, genes encoding key components of the vitamin D pathway were chosen, which include: enzymes responsible for the activation and inactivation vitamin D (CYP27A1, CYP27B1, CYP2R1 and CYP24A1) [31,32]; the vitamin D binding protein (GC) that binds to vitamin D and its plasma metabolites and transports them to target tissues; SKIIP, also known as NCoA62/SKIP, that serves as a coactivator a vitamin D-mediated transcription [33]; and five revevant genes for asthma that are known to be transcriptionally regulated by vitamin D (*IL10*, *IL1RL1*, *CD28*, *CD86 *and *IL8*) [14].

### SNP selection

SNPs were selected using the CEPH genotype dataset from phase 1 of the International HapMap project [34]. The genotype data were downloaded from the genomic region covering ten kilobases up- and downstream of each gene. A maximally informative set of SNPs was selected using a pairwise tagging algorithm described by Carlson et al. [35]. A Perl program, called ldSelect http://droog.gs.washington.edu/ldSelect.html, was used to select the SNPs in each gene. Briefly, this program analyzes the pattern of LD between SNPs and forms bins of SNPs in LD based on an r^2 ^threshold. The algorithm ensures that all pairwise LD values between SNPs in the same bin exceed the r^2 ^threshold. Accordingly, any SNP in a bin can serve as a proxy (tagSNP) for all other SNPs in the same bin. Only one tagSNP needs to be typed per bin. At this level, nonsynonymous SNPs genotyped in the HapMap dataset were prioritized using the "-required" option. Similarly, some SNPs were prioritized based on the type of variation (A/T, C/T, etc) to meet the genotyping technology requirement. The minor allele frequency and the r^2 ^thresholds were set at 0.05 and 0.8, respectively, using the "-freq" and "-r2" options. Known nonsynonymous SNPs or functional variants not genotyped in the HapMap dataset were also selected for genotyping. Selected SNPs and their characteristics are shown in additional file (see Additional file 1). The location of SNPs relative to the gene structure is illustrated in Additional file 2.

### Genotyping

In the SLSJ study, a total of 87 SNPs were genotyped using the SNP stream^® ^UHT technology [36]. Primers were designed using FastPCR version 3.8.78 for multiplex PCR [37]. Single base extension primers were designed using Autoprimer.com (Beckman Coulter). The protocol and reaction conditions were performed in accordance with the manufacturer [36]. SNPs were genotyped in different panels that were organized by grouping SNPs with the same type of variation (A/T, C/T, etc) and by respecting the 12-plex maximum capacity of the system. For the replication studies (CAPPS, SAGE and BHS), 52 SNPs located in five genes were genotyped using the Illumina GoldenGate assay [38] as part of a larger SNP genotyping panel http://www.genapha.ca. SNP genotypes in CAMP available from prior analyses were generated for VDR [22] and IL10 [39] using the MassARRAY platform (Sequenom, San Diego, CA) and SNaPShot (Applied Biosystems, Forrest City, CA), respectively, as previously described.

### Statistical analyses

Mendelian inheritance incompatibilities were inspected using Pedmanager version 0.9 and Hardy-Weinberg equilibrium was evaluated using a χ^2 ^test among parents. For the SLSJ, CAPPS and SAGE studies the Family Based Association Test (FBAT) program was used to test association with single SNPs [40]. All tests were performed with an additive model using the empirical variance-covariance estimator that adjusts for the correlation among sibling genotypes and for multiple nuclear families within a single pedigree. The FBAT test provides a Z-statistic with the corresponding p value. A positive Z-statistic is indicative of a high-risk allele and a negative Z-statistic is indicative of a protective allele. In the BHS, the single SNP associations were evaluated using the Cochran-Armitage test for trend with additive coding of alleles. Genes showing at least one SNP with a p < 0.05 in the SLSJ collection were considered for validation in the other populations. Our strategy to deal with multiple testing was to replicate the associations in independent populations instead of using an adjusted p value. LD values were evaluated using the r^2 ^metrics and calculated with Haploview 3.32 [41]. Power calculations for the four study populations were recently described [42].

Gene-gene interactions were evaluated for asthma and atopy using a multilocus analysis method following the framework described in Millstein et al. [23]. This strategy is based on likelihood-ratio tests that used a log-additive coding scheme, where genotypes aa, Aa, and AA are coded as 0, 1, and 2, respectively. Briefly, the analyses were performed in two stages. In stage one, single SNP tests of associations were evaluated by contrasting the null hypothesis of no association with the alternative hypothesis. The threshold for significance for stage one of the gene-gene interaction tests was then adjusted for multiple testing using the Bonferroni correction. In stage two, a full two-gene interaction model, including the two main effects and the interaction term, was tested against the reduced model that includes only the main effects that were declared significant in the first stage, if any. This strategy avoids retesting the same effects detected in stage one. While the framework of Millstein et al. [23] was described for a case-control dataset, it can easily be adapted to a case-parent design by following the case/pseudocontrol design described by Cordell et al. [43], where each case is matched with three pseudocontrols derived from the untransmitted parental alleles. Following this, conditional logistic regression is used to assess the significance of the main and interaction terms. Since transmission to multiple affected siblings cannot be assumed to be independent events, and since the families in our sample may contain more than one case, robust estimates for the variance and Wald tests were used instead of likelihood ratio tests for the SLSJ, CAPPS and SAGE studies. Considering the number of SNPs genotyped in genes involved in the vitamin D pathway, a total of 5003 two-gene interaction models were evaluated.

*Post hoc *analyses were performed with the combined dataset (SLSJ, CAPPS, SAGE, and BHS). Tests of association were performed using the likelihood method implemented in UNPHASE v3.0.10 [44], which allow data from family studies and case-control individuals to be analyzed together.

## Results

### Results from the SLSJ population

Additional file 1 presents the 87 genotyped SNPs and their characteristics in the SLSJ study. Two SNPs failed the assay design including one in the *CD86 *gene (rs1915087) and another in the *GC *gene (rs1491711). Both of these SNPs are singletons and are not tagging other SNPs in the genes. A third SNP (rs8176353) located in the *CYP27B1 *gene was monomorphic. Additional file 1 also showed the minor allele frequencies for a reference population (CEPH from HapMap) and for the SLSJ study. In most cases, the minor allele frequencies were very similar between the two populations with a mean difference of 2% and the largest difference was 16% for SNP rs4308217 located in the *CD86 *gene. After Bonferroni correction, only one SNP was out of Hardy-Weinberg equilibrium (see Additional file 1). This SNP (rs4809960) is a singleton in the *CYP24A1 *gene and was removed from further analyses. Accordingly, a total of 83 SNPs were tested for association with asthma and atopy. Additional file 2 shows the exon-intron structure of each gene and the location of genotyped SNPs.

**Figure 2:**
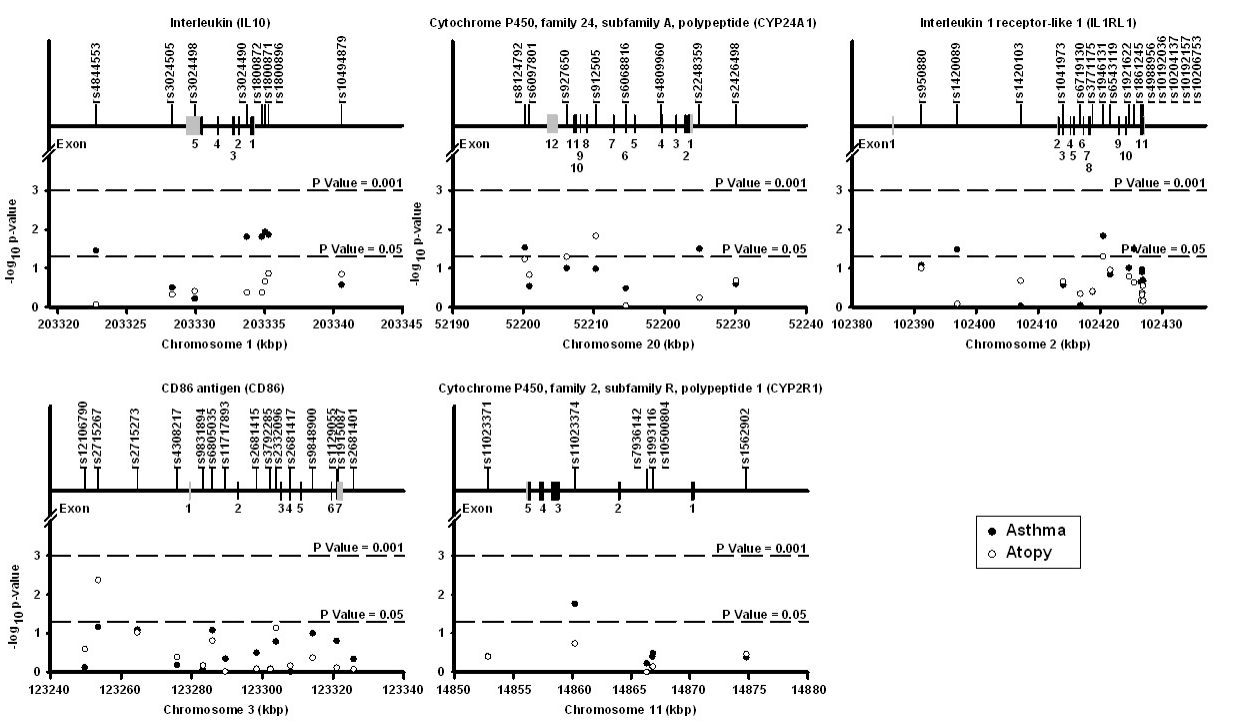
**Genetic association of SNPs in the vitamin D pathway genes with asthma and atopy in the SLSJ study**. Only genes with a least one significant p value (p < 0.05) are illustrated. Each subfigure presents the result of one gene. The top line indicates the gene name and symbol. The upper part of each subfigure shows the exon-intron structure of the gene and the localization of the genotyped SNPs. The coding exons are shown in black and the untranslated regions are shown in grey. The lower part of each subfigure illustrates the association results for asthma (solid circles) and atopy (open circles). The x-axis shows the localization of the gene and SNPs on NCBI Human Genome build 35. The y-axis shows the FBAT empirical p values on a log_10 _scale. The lower and upper dashed lines represent p value thresholds of 0.05 and 0.001, respectively. The upper and lower parts of each subfigure are shown on the same scale.

**Figure 3:**
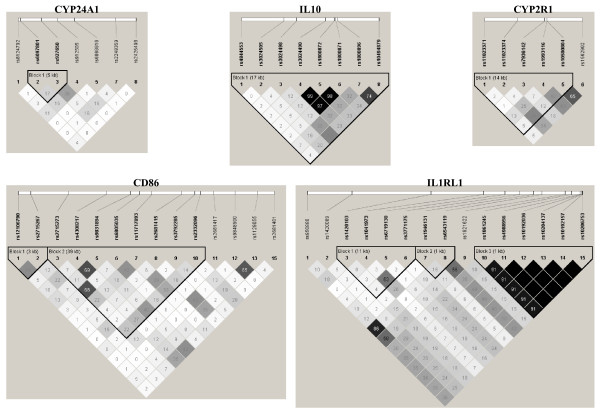
**Linkage disequilibrium (LD) plots surrounding five genes involved in the vitamin D pathway in the SLSJ study**. The LD plots were generated by Haploview 3.32 [41]. Gene symbols are indicated at the top of each graph. The top horizontal bar illustrates the location of SNPs on a physical scale. The color of squares illustrates the strength of pairwise r^2 ^values on a black and white scale where black indicates perfect LD (r^2 ^= 1.00) and white indicates perfect equilibrium (r^2 ^= 0). The r^2 ^LD value is also indicated within each square. Blocks are defined using the Gabriel et al [71] definition. Failed and monomorphic SNPs as well as SNPs not in Hardy-Weinberg equilibrium are not illustrated.

The overall distribution of single marker FBAT association tests shows a greater number of small p values for asthma compared to what was expected by chance (see Additional file 3). Results for genes with at least one significant p value for asthma and atopy are illustrated in Figure 2 (results for all genes are illustrated in Additional file 2). The details of these tests are shown in Table 2 for SNPs having at least one p value < 0.1 for asthma or atopy (FBAT results for all SNPs can be found in Additional file 4). Five SNPs in the *IL10 *gene, three located in the promoter (rs1800872, rs1800871, rs1800896), one in intron 1 (rs3024490), and the other located in the 3' region (rs4844553) were significantly associated with asthma. Three of them (rs1800871, rs1800872, and rs3024490) were in tight LD (r^2 ^> 0.97), while the others were in low to modest LD (Figure 3). Haplotype analysis for the three tightly linked SNPs revealed the presence of only two haplotypes with an allele frequency above 1% in the SLSJ population. The TAA haplotype had a frequency of 0.268 and was overtransmitted to asthma patients (p = 0.024), while the CCC haplotype had a frequency of 0.726 and was undertransmitted (p = 0.013). Four out of the eight genotyped SNPs in the *CYP24A1 *gene were also modestly associated with asthma or atopy (range of p values = 0.051 to 0.015). The two intronic SNPs associated with atopy (rs912505 and rs927650) were in modest LD (r^2 ^= 0.36) and the two SNPs associated with asthma (rs2248359 and rs8124792) located in the promoter and the 3' region of the gene were in complete equilibrium (r^2 ^= 0) (Figure 3). Three intronic SNPs in the *IL1RL1 *gene were also associated with asthma. Two of them (rs1420089 and rs1861245) were in modest LD (r^2 ^= 0.28) and the third one (rs1946131) showed no LD with the other (Figure 3). Also worth mentioning is a SNP (rs2715267) in the promoter region of the *CD86 *gene that was significantly associated with atopy (p = 0.004). A trend for this SNP was also observed for asthma (p = 0.069). Finally, one SNP (rs11023374) in intron 2 of the *CYP2R1 *gene was associated with asthma (p = 0.017). LD plots for all genes are illustrated in Additional file 5.

**Table 2:** Single SNP association results for asthma and atopy in the Saguenay^_^Lac-Saint-Jean study.

		Asthma					Atopy			
			
Gene	SNP	Allele	Allele frequency	# of families*	Z	P value	Allele frequency	# of families*	Z	P value
*IL10*	rs4844553	C	0.94	39	2.10	**0.036**	0.94	33	0.15	0.881
		T	0.06	39	-2.10	**0.036**	0.06	33	-0.15	0.881
	rs3024490	A	0.28	105	2.42	**0.016**	0.28	94	0.80	0.423
		C	0.72	105	-2.42	**0.016**	0.72	94	-0.80	0.423
	rs1800872	A	0.28	105	2.42	**0.016**	0.28	94	0.80	0.423
		C	0.72	105	-2.42	**0.016**	0.72	94	-0.80	0.423
	rs1800871	C	0.71	95	-2.52	**0.012**	0.71	84	-1.22	0.221
		T	0.29	95	2.52	**0.012**	0.29	84	1.22	0.221
	rs1800896	C	0.46	117	-2.47	**0.014**	0.46	101	-1.49	0.137
		T	0.54	117	2.47	**0.014**	0.54	101	1.49	0.137

*IL1RL1*	rs950880	A	0.39	126	1.73	0.084	0.39	105	1.64	0.100
		C	0.61	126	-1.73	0.084	0.61	105	-1.64	0.100
	rs1420089	C	0.18	72	-2.14	**0.033**	0.18	60	-0.21	0.832
		T	0.82	72	2.14	**0.033**	0.82	60	0.21	0.832
	rs1946131	C	0.92	53	-2.44	**0.015**	0.92	42	-1.96	**0.050**
		T	0.09	53	2.44	**0.015**	0.09	42	1.96	**0.050**
	rs1921622	C	0.54	75	-1.65	0.099	0.54	65	-1.39	0.165
		T	0.46	75	1.65	0.099	0.46	65	1.39	0.165
	rs1861245	A	0.43	101	-2.14	**0.032**	0.43	74	-1.19	0.235
		G	0.57	101	2.14	**0.032**	0.57	74	1.19	0.235

*CD28*	rs6435203	C	0.34	120	-1.97	**0.049**	0.34	104	-1.17	0.243
		T	0.67	120	1.97	**0.049**	0.67	104	1.17	0.243

*CYP27A1*	rs4674338	C	0.60	96	1.85	0.064	0.60	82	1.72	0.085
		T	0.41	96	-1.85	0.064	0.41	82	-1.72	0.085

*CD86*	rs2715267	A	0.64	102	-1.82	0.069	0.64	91	-2.86	**0.004**
		C	0.37	102	1.82	0.069	0.37	91	2.86	**0.004**
	rs2715273	A	0.82	79	1.75	0.081	0.82	65	1.67	0.096
		T	0.18	79	-1.75	0.081	0.18	65	-1.67	0.096
	rs6805035	A	0.88	51	1.74	0.083	0.88	43	1.43	0.154
		C	0.12	51	-1.74	0.083	0.12	43	-1.43	0.154
	rs2332096	A	0.46	117	-1.39	0.164	0.46	108	-1.79	0.074
		C	0.54	117	1.39	0.164	0.54	108	1.79	0.074

*CYP2R1*	rs11023374	C	0.33	105	2.38	**0.017**	0.33	88	1.33	0.183
		T	0.67	105	-2.38	**0.017**	0.67	88	-1.33	0.183

*CYP24A1*	rs8124792	C	0.95	22	2.18	**0.030**	0.95	24	1.89	0.058
		T	0.05	22	-2.18	**0.030**	0.05	24	-1.89	0.058
	rs927650	C	0.55	108	-1.65	0.100	0.55	91	-1.95	0.051
		T	0.45	108	1.65	0.100	0.45	91	1.95	0.051
	rs912505	C	0.29	89	-1.63	0.104	0.29	80	-2.44	**0.015**
		T	0.71	89	1.63	0.104	0.71	80	2.44	**0.015**
	rs2248359	C	0.58	112	2.15	**0.032**	0.58	97	0.56	0.577
		T	0.42	112	-2.15	**0.032**	0.42	97	-0.56	0.577

*Number of informative families to conduct the test.

P values < 0.05 are shown in bold.

**Figure 4:**
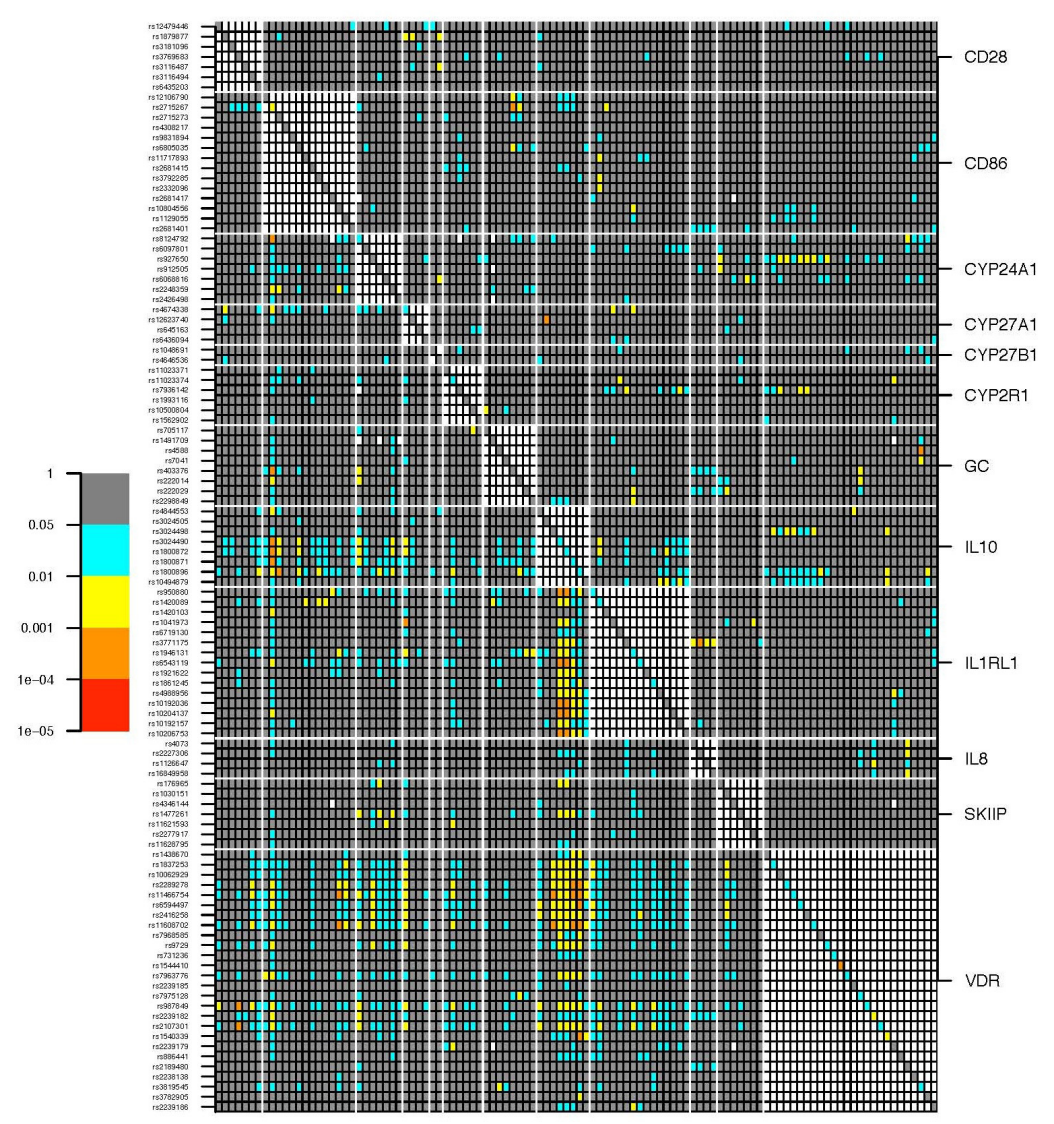
**Two-gene model analyses on asthma in the SLSJ study for genes involved in the vitamin D pathway**. The plot illustrates the p values for single SNP association and all possible two-SNP combinations. Each line represents a SNP listed at the left of the figure. Each column represents a SNP listed in the same order but from left to right. The white horizontal and vertical lines break up the figure by genes. Gene names are indicated at the right of the figure. P values are illustrated using the following color scheme: grey, p > 0.05; blue, 0.05 > p > 0.01; yellow, 0.01 > p > 0.001; orange, 0.001 > p > 0.0001, red, 0.0001 > p > 0.00001. The squares forming the diagonal (upper-left to lower-right) depict p values for single SNP association based on a Wald test (see materials and methods). Two-gene models for SNPs located in the same gene were not assessed and were not coloured, which created the large white square patterns along the diagonal. Illustrated above the diagonal are p values for the interaction term only, which are the results of Wald tests contrasting the full model (two main effects and the interaction) to the reduced model (two main effects only). Illustrated below the diagonal are p values testing the full model (two main effects and the interaction) against a reduced model that is conditional on single SNPs declared significant when taken individually, if any (see materials and methods). White squares illustrate tests for which the model failed to converge.

Interaction among functionally related genes may not be surprising. Hence, all possible two-gene interactions were tested for asthma in the SLSJ study for the 11 genes under study plus the *VDR *gene (Figure 4). Two concentrated spots of significant two-gene models for asthma are observed in this figure. The one at the bottom represents two-gene models involving SNPs in the *IL10 *and *VDR *genes. The second spot located in the center of Figure 4 represents two-gene models involving SNPs in the *IL10 *and *IL1RL1 *genes. Multiple two-gene models were also significant between SNPs in the *IL10 *and *CD86 *genes. Additional file 6 shows the two-gene models for atopy in the SLSJ study. Overall, gene-gene interactions were modest for atopy.

**Figure 5:**
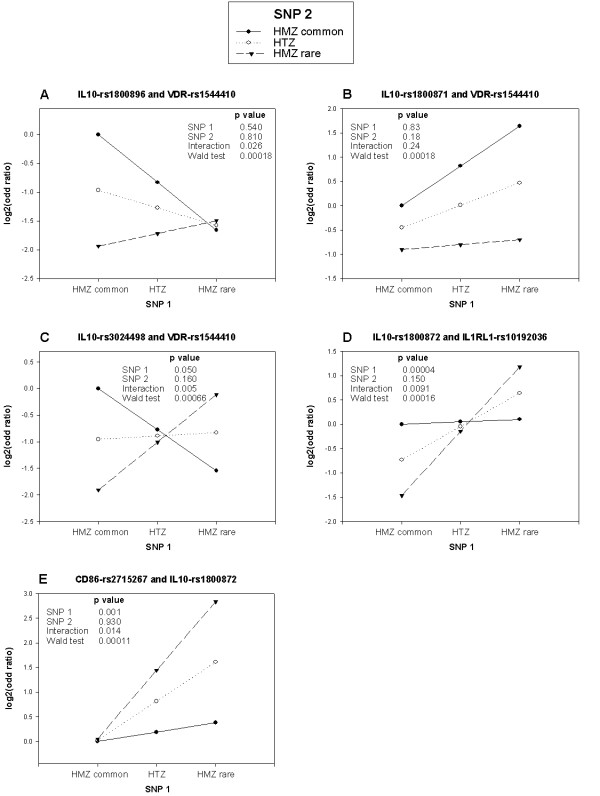
**Combined genotypic effects of selected two-gene models on asthma in the SLSJ study**. Each subfigure illustrates the risk of asthma according to two SNPs located in different genes. The genes and SNPs named are indicated above each subfigure representing SNP 1 and SNP 2, respectively. With two bi-allelic SNPs (3 genotypes per SNP), a 3 by 3 odds ratio matrix is calculated and visually represented. All risks are evaluated relative to homozygotes for the common allele at both SNPs. The y-axis shows the odds ratio on a log_2 _scale, which makes the odds ratio above and below 1 on the same visual scale. The x-axis indicates the genotypes for SNP number 1. By drawing a line that joins the dots representing the genotypes for SNP number 2, the genotypic effect of SNP number 1 can be observed on different genotypic backgrounds of SNP number 2. The first three p values come from a model testing the main effect of both SNPs and the interaction term. The Wald test p value is the result of the full two-gene model tested against a reduced model (see materials and methods).

To understand the impact of these two-gene models on the risk of asthma, the genotype by genotype odds ratio matrix was calculated and some representative and most significant two-gene models are illustrated in Figure 5. Figure 5a, b and 5c show two-gene models between SNPs in the *IL10 *and *VDR *genes. Figure 5a shows that the risk of having asthma is similar for carriers of two rare *IL10 *alleles irrespective of the *VDR *genotypes. However, the risk increases with the increasing number of common *IL10 *alleles for individuals who are homozygous for the common *VDR *allele. In contrast, the risk tends to decrease with the increasing number of common *IL10 *alleles for individuals who are homozygous for the rare *VDR *allele. Figure 5b shows that the risk of asthma increases with the number of rare *IL10 *alleles, but the effect is greater with an increasing number of common alleles at the *VDR *locus. Figure 5a and 5b show representative interactions between SNPs located in the promoter region of *IL10 *and the 3'UTR region of *VDR*. However, more complex interactions between these two genes were observed between SNPs located in the 3'UTR region of both genes. Figure 5c shows that the effect of the *IL10 *rare allele goes in the opposite directions depending on whether subjects are homozygous for the common or the rare *VDR *alleles. Figure 5d shows a representative interaction between promoter polymorphisms in the *IL10 *gene and nonsynonymous SNPs located in the *IL1RL1 *gene. In this model, the rare *IL10 *alleles increase the risk, but the magnitude of the effect is greater with the number of rare alleles at the *IL1RL1 *gene. Figure 5e shows a representative interaction between promoter polymorphisms in the *IL10 *and *CD86 *genes. In this model, the risk of asthma is similar for carriers of two common *CD86 *alleles irrespective of the *IL10 *genotypes, however, the risk increases additively with the number of rare alleles in the two genes.

### Replication samples

For all the single SNP associations observed in the SLSJ population, the statistical significance was modest and did not survive multiple correction procedures. Accordingly, an effort was made to replicate these findings in three additional studies. A comprehensive set of tagging SNPs in the *IL10*, *CYP24A1*, *IL1RL1*, *CD86*, and *CYP2R1 *genes plus the significant (p < 0.05) SNPs in the SLSJ study were genotyped in the CAPPS, SAGE and BHS studies. Table 3 shows SNPs with at least one p value < 0.05 for asthma or atopy in the three studies as well as SNPs with p value < 0.05 in the SLSJ study. Complete results for the replicate samples can be found in Additional file 7. For *IL10*, none of the SNPs associated with asthma in the SLSJ study were significant in the other studies except the promoter polymorphism rs1800896, with a marginal p value of 0.022 in CAPPS. However, the C allele that was protective in the SLSJ study was the risk allele in the CAPPS study. The direction of the risk allele in the SAGE study was similar to the one observed in the SLSJ, but the effect was not significant. Similar flip-flop phenomena were observed between the SAGE and CAPPS studies for SNPs that were not significant in the SLSJ study. Two SNPs in tight LD, rs3024498 located in the 3'UTR and rs3024492 located in intron 3, showed p values > 0.01 in both replicate samples, but the orientation of the risk allele was reversed. For most *IL10 *SNPs, it was noticeable that the orientation of the risk alleles is in agreement between the SLSJ and SAGE studies and in the opposite direction for CAPPS. No SNP in the *IL10 *gene was significantly associated with asthma or atopy in the BHS. Taken together, significant associations in the *IL10 *gene were observed in three studies. However, different SNPs were associated in different populations and the direction of the risk allele in the CAPPS study was reversed compared with the SLSJ and SAGE studies.

**Table 3:** Single SNP association results for asthma and atopy in the replication samples.

			Asthma												Atopy											
				
			SAGE				CAPPS				BHS				SAGE				CAPPS				BHS			
					
Genes	SNPs	Allele	AF	Fa	Z	p	AF	Fa	Z	p	AF case	AF ctrl	X^2^	p	AF	Fa	Z	p	AF	Fa	Z	p	AF case	AF ctrl	X^2^	p
*IL10*	rs4844553	A	0.07	25	0.96	0.336	0.05	11	0.3	0.763	0.05	0.06	1.21	0.272	0.07	35	-0.16	0.873	0.05	22	-1.88	0.061	0.05	0.06	0.12	0.729
		G	0.93	25	-0.96	0.336	0.95	11	-0.3	0.763					0.93	35	0.16	0.873	0.95	22	1.88	0.061				
	rs3024498	A	0.78	83	3.06	**0.002**	0.78	40	-2.56	**0.01**	0.70	0.72	1.74	0.187	0.78	108	1.22	0.223	0.78	70	-0.54	0.592	0.71	0.72	0.26	0.608
		G	0.23	83	-3.06	**0.002**	0.22	40	2.56	**0.01**					0.23	108	-1.22	0.223	0.22	70	0.54	0.592				
	rs3024492	A	0.78	81	2.89	**0.004**	0.78	40	-2.56	**0.01**	0.70	0.72	1.55	0.213	0.78	108	1.13	0.26	0.78	69	-0.75	0.453	0.71	0.72	0.20	0.655
		T	0.22	81	-2.89	**0.004**	0.22	40	2.56	**0.01**					0.22	108	-1.13	0.26	0.22	69	0.75	0.453				
	rs3024490	A	0.28	69	0.63	0.527	0.29	34	-1.64	0.101	0.24	0.22	1.63	0.202	0.28	99	0.36	0.717	0.29	78	0	1	0.23	0.23	0.07	0.788
		C	0.72	69	-0.63	0.527	0.71	34	1.64	0.101					0.72	99	-0.36	0.717	0.71	78	0	1				
	rs1800872	A	0.28	70	0.73	0.463	0.3	34	-1.64	0.101	0.24	0.22	1.64	0.200	0.28	98	0.46	0.649	0.3	78	-0.1	0.922	0.23	0.23	0.07	0.786
		C	0.72	70	-0.73	0.463	0.7	34	1.64	0.101					0.72	98	-0.46	0.649	0.7	78	0.1	0.922				
	rs1800896	C	0.43	87	-1.22	0.221	0.43	40	2.29	**0.022**	0.50	0.50	0.02	0.889	0.43	124	-0.55	0.583	0.43	83	0.29	0.772	0.50	0.50	0.06	0.804
		T	0.57	87	1.22	0.221	0.58	40	-2.29	**0.022**					0.57	124	0.55	0.583	0.58	83	-0.29	0.772				
	rs10494879	C	0.63	93	1.44	0.151	0.64	42	-2.07	**0.039**	0.55	0.56	0.51	0.476	0.63	127	0.08	0.938	0.64	82	-1.18	0.239	0.55	0.56	0.13	0.717
		G	0.37	93	-1.44	0.151	0.36	42	2.07	**0.039**					0.37	127	-0.08	0.938	0.36	82	1.18	0.239				

*IL1RL1*	rs1420089	C	0.09	39	1.41	0.16	0.09	19	-0.23	0.819	0.11	0.11	0.08	0.782	0.09	50	1.91	0.057	0.09	40	0	1	0.12	0.12	0.03	0.874
		T	0.91	39	-1.41	0.16	0.91	19	0.23	0.819					0.91	50	-1.91	0.057	0.91	40	0	1				
	**rs1041973**	A	0.22	80	-0.1	0.921	0.23	35	0.16	0.876					0.22	98	-1.99	**0.046**	0.23	73	0.11	0.915				
		C	0.78	80	0.1	0.921	0.77	35	-0.16	0.876					0.78	98	1.99	**0.046**	0.77	73	-0.11	0.915				
	rs1946131	A	0.11	43	-0.3	0.768	0.1	16	-0.23	0.819	0.10	0.10	0.36	0.55	0.11	52	-1.05	0.294	0.1	37	0.78	0.435	0.10	0.09	1.61	0.205
		G	0.9	43	0.3	0.768	0.9	16	0.23	0.819					0.9	52	1.05	0.294	0.9	37	-0.78	0.435				

*CD86*	rs9282641	C	0.93	36	0.8	0.423	0.93	14	-0.78	0.439	0.89	0.92	7.34	**0.007**	0.93	42	0	1	0.93	35	-0.16	0.873	0.91	0.90	0.82	0.365
		T	0.07	36	-0.8	0.423	0.07	14	0.78	0.439					0.07	42	0	1	0.07	35	0.16	0.873				
	rs9831894	G	0.42	88	-1.12	0.261	0.4	40	-2.61	**0.009**	0.39	0.39	0.00	0.952	0.42	124	-0.23	0.817	0.4	96	-0.26	0.792	0.39	0.40	0.21	0.645
		T	0.58	88	1.12	0.261	0.6	40	2.61	**0.009**					0.58	124	0.23	0.817	0.6	96	0.26	0.792				
	rs2332096	G	0.57	87	-1.53	0.127	0.56	43	-2.36	**0.018**	0.53	0.55	0.47	0.491	0.57	129	-1.08	0.28	0.56	94	-0.92	0.357	0.54	0.54	0.00	0.958
		T	0.44	87	1.53	0.127	0.44	43	2.36	**0.018**					0.44	129	1.08	0.28	0.44	94	0.92	0.357				

*CYP2R1*	rs11023374	C	0.27	72	-0.63	0.532	0.27	35	0.44	0.662	0.27	0.28	0.07	0.793	0.27	91	0.38	0.703	0.27	72	1.05	0.292	0.28	0.27	0.08	0.775
		T	0.73	72	0.63	0.532	0.73	35	-0.44	0.662					0.73	91	-0.38	0.703	0.73	72	-1.05	0.292				
	rs10500804	G	0.4	92	1.81	0.07	0.44	40	-0.41	0.68	0.41	0.44	4.25	**0.039**	0.4	119	0.72	0.47	0.44	94	0.81	0.421	0.42	0.43	0.30	0.582
		T	0.6	92	-1.81	0.07	0.56	40	0.41	0.68					0.6	119	-0.72	0.47	0.56	94	-0.81	0.421				
	rs1562902	C	0.46	93	-0.83	0.405	0.42	39	-0.42	0.674	0.47	0.43	4.71	**0.03**	0.46	126	0.16	0.874	0.42	93	-0.62	0.538	0.46	0.43	2.38	0.123
		T	0.54	93	0.83	0.405	0.58	39	0.42	0.674					0.54	126	-0.16	0.874	0.58	93	0.62	0.538				

*CYP24A1*	rs8124792	A	0.06	30	0.17	0.862	0.08	12	-0.26	0.796	0.06	0.06	0.29	0.591	0.06	42	1.18	0.238	0.08	40	0.15	0.881	0.07	0.05	4.65	**0.031**
		G	0.94	30	-0.17	0.862	0.93	12	0.26	0.796					0.94	42	-1.18	0.238	0.93	40	-0.15	0.881				
	rs927650	C	0.54	94	0.81	0.417	0.57	41	-0.69	0.492	0.55	0.50	6.75	**0.009**	0.54	127	0.23	0.816	0.57	100	0.78	0.439	0.54	0.52	0.89	0.344
		T	0.46	94	-0.81	0.417	0.43	41	0.69	0.492					0.46	127	-0.23	0.816	0.43	100	-0.78	0.439				
	rs912505	A	0.75	75	0.53	0.596	0.73	29	0.69	0.493	0.78	0.79	0.41	0.521	0.75	106	0.83	0.409	0.73	86	-0.2	0.843	0.77	0.79	1.64	0.200
		G	0.25	75	-0.53	0.596	0.27	29	-0.69	0.493					0.25	106	-0.83	0.409	0.27	86	0.2	0.843				
	rs2248359	A	0.35	87	1.04	0.296	0.44	41	1.82	0.069	0.41	0.41	0.02	0.889	0.35	133	0.54	0.588	0.44	97	2.51	**0.012**	0.42	0.40	1.14	0.287
		G	0.65	87	-1.04	0.296	0.56	41	-1.82	0.069					0.65	133	-0.54	0.588	0.56	97	-2.51	**0.012**				
	rs2426498	C	0.87	49	-1.39	0.166	0.87	24	0.82	0.414	0.86	0.87	0.03	0.859	0.87	58	-2.14	**0.032**	0.87	61	0.25	0.806	0.86	0.87	0.06	0.814
		G	0.13	49	1.39	0.166	0.13	24	-0.82	0.414					0.13	58	2.14	**0.032**	0.13	61	-0.25	0.806				
	rs6068821	A	0.41	95	2.32	**0.021**	0.45	39	0.27	0.785	0.41	0.39	1.15	0.283	0.41	126	2.24	**0.025**	0.45	96	1.17	0.241	0.42	0.40	1.07	0.302
		G	0.59	95	-2.32	**0.021**	0.55	39	-0.27	0.785					0.59	126	-2.24	**0.025**	0.55	96	-1.17	0.241				

CAPPS, Canadian Asthma Primary Prevention Study; SAGE, Study of Asthma Genes and the Environment; BHS, Busselton Health Study; AF, allele frequency; Fa, Number of informative families to conduct the test.

P values below 0.05 are shown in bold. SNPs that are significant in the SLSJ study (Table 2) are underlined as well as the allele that increases the risk in that study. Coding non-synonymous SNPs are shown in bold.

The associations observed for the *IL1RL1 *and *CYP2R1 *genes in the SLSJ study were not validated in the replication samples (Table 3). No other SNP in the *IL1RL1 *gene was associated with asthma or atopy in the replication studies except a non-synonymous coding SNP (rs1041973, Glu78Ala) that was borderline significant for atopy in SAGE. For *CYP2R1*, two additional SNPs located in the promoter and intron 1 were associated with asthma in the BHS. However, none of these SNPs were replicated in the other studies. For *CD86*, the promoter SNP (rs2715267) that was associated with atopy in the SLSJ (p = 0.004) could not be assessed in the other studies due to failure of the genotyping assay. However, two other intronic SNPs (rs9831894 and rs2332096, r^2 ^= 0.48 in the European HapMap data), not in LD with rs2715267, were significantly associated with asthma in CAPPS. A different SNP (rs9282641) located in non-coding exon 1 was significantly associated with asthma in the BHS. This latter SNP was not in LD with any of the three SNPs associated with asthma in CAPPS and SLSJ. Accordingly, different *CD86 *polymorphims were associated with asthma in three populations. Finally, significant SNPs were observed in the *CYP24A1 *gene in all studies. However, there was no consistency with the SLSJ study in terms of phenotype and orientation of the risk alleles.

The two-gene models were also performed in the replication samples for the five genes that were genotyped in CAMP, SAGE and BHS. The significant two-gene models involving *IL10 *and *VDR *as well as *IL10 *and *IL1RL1 *observed in the SLSJ study is not replicated in the CAPPS, SAGE and BHS. It should be noted that the sample sizes in the SAGE and CAPPS studies limit our ability to replicate the findings. For example, the number of cases contributing to the two-gene model involving *IL10*-rs1800896 and *VDR*-rs1544410 (Figure 5a) was approximately halved in SAGE and reduced to a quarter in CAPPS compared with the SLSJ study. However, even with similar power, the significant two-gene models observed in the SLSJ are not replicated in the BHS (see Additional file 8). Taken together, none of the significant two-gene models observed in the SLSJ study were properly replicated in CAPPS, SAGE and BHS.

**Figure 6:**
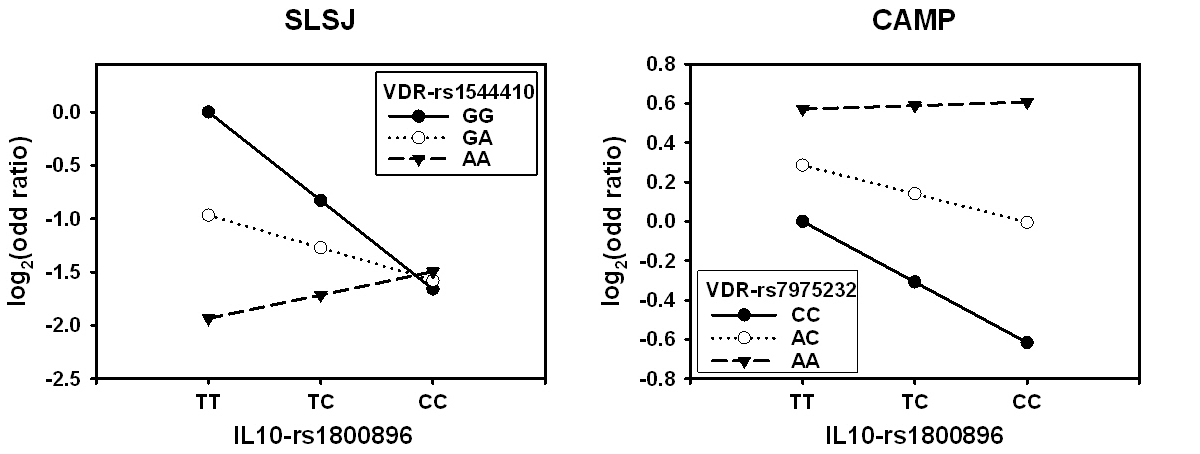
**Two-gene models on asthma involving the *IL10 *and *VDR *genes in the SLSJ and the CAMP studies**. With two bi-allelic SNPs (3 genotypes per SNP), a 3 by 3 odds ratio matrix is calculated and visually represented. The y-axis shows the odds ratio on a log_2 _scale, which makes the odds ratio above and below 1 on the same visual scale. The *IL10 *genotypes are illustrated on the x-axis. Different panels of SNPs were genotyped between the two studies and the comparison for the *VDR *gene is made with different SNPs that are in LD (D' = 1.0 and r^2 ^= 0.59, based on the HapMap CEU genotyping dataset). In the SLSJ study, the risks are evaluated relative to the *VDR*-GG and *IL10*-TT genotypes. In CAMP, the risks are evaluated relative to the *VDR*-CC and *IL10*-TT genotypes.

The significant two-gene models involving *IL10 *and *VDR *were also tested in the CAMP study. A different panel of SNPs was genotyped in CAMP and some of the two-gene models are significant (p < 0.05). The most significant two-gene models is observed between *VDR*-rs7975232 and *IL10*-rs1800896 (Wald test p = 0.018) or *IL10*-rs1800872 (Wald-test p = 0.016). Figure 6 shows the comparison of two-gene models observed in the SLSJ and CAMP studies. *VDR*-rs1544410 is not genotyped in CAMP, but rs7975232 was in moderately high LD with it (D' = 1.0 and r^2 ^= 0.59, based on the HapMap CEU genotyping dataset). The gene-gene interaction models were similar between the two studies. The effect of the *IL10 *variant on asthma seemed dependent on the *VDR *variant. This can be appreciated in Figure 6 by comparing the solid, dotted and dashed lines between studies. The flip-flop phenomenon previously observed in the *VDR *gene between the SLSJ and CAMP studies [21,22] can also be appreciated in Figure 6 (the solid line is at the top in the SLSJ study and at the bottom in the CAMP study).

### Combined analyses

Single SNP association tests and two-gene models were tested in the combined dataset. Overall, none of the SNPs are significantly associated with asthma or atopy after corrections for multiple testing (see Additional file 9). Similarly, the two-gene models for asthma and atopy were not significant (see Additional files 10 and 11, respectively).

## Discussion

We performed a genetic association study between asthma and genes involved in the vitamin D pathway. SNPs located in five genes, including *IL10*, *CYP24A1*, *IL1RL1*, *CYP2R1 *and *CD86*, showed modest association with asthma or atopy in an asthma family study derived from the Saguenay^_^Lac-Saint-Jean population. Exploring gene-gene interactions in this pathway revealed significant two-gene models that refined risk evaluation for asthma. In an attempt to replicate these findings, we evaluated the significant genes in two additional Canadian studies and one Australian study. Again modest associations were observed for the *IL10*, *CYP24A1 *and *CD86 *genes for asthma or atopy. In general, SNPs showing association were different between studies or the orientation of the risk allele was reversed. Similarly, the significant two-gene models found in the original study were not replicated in the two additional Canadian studies and the Australian study. However, a significant two-gene model involving the *IL10 *and *VDR *gene was replicated in the American study.

Overall, no genes or gene-gene interactions in the vitamin D pathway were consistently associated with asthma or atopy across all populations. This can be explained by multiple factors as discussed previously [3,42]. Briefly, asthma was defined differently between populations, including patient self-reported, physician's diagnosis, or a combination of clinical characteristics and objective threshold from methacholine challenges. In addition, the individual studies are either early or late-onset asthma. More specific for the current study, many environmental factors such as sun exposure and dietary vitamin D intake may differ between studies and explained some of the controversies and flip-flop observations. Considering the differences among populations, our replication effort may be more properly labeled as a descriptive study.

A number of SNPs in the *IL10 *gene surrounding the promoter region and extending up to intron 1 were significantly associated with asthma in the SLSJ collection. All these SNPs were in tight LD and most of the genetic diversity at this locus was captured by only two haplotypes. Based on functional studies [45-50], the three-SNP promoter haplotypes formed by rs180096, rs1800871, and rs1800872 have been referred as the high and low IL10-producing haplotypes. IL10 is known as an anti-inflammatory cytokine [51]. Accordingly, it makes biological sense that a low IL10-producing haplotype would be associated with asthma, which is consistent with most of the literature [39,52-54]. Surprisingly, none of the promoter polymorphisms in IL10 were associated with asthma or atopy in the SAGE and CAPPS populations with the exception of rs1800896, which was associated with asthma in CAPPS. However, the orientation of the risk allele was reversed compared to what was observed in the SLSJ study and the vast majority of the scientific literature. In contrast, SNPs located in the 3' end of the *IL10 *gene (rs3024498 and rs3024492) were associated with asthma in the replicate samples. The functional consequences of these SNPs are unknown but they were previously associated with serum levels of calcidiol (25-OH-D_3_), the prehormone form of vitamin D [55], and with longitudinal decline rate of lung function [56].

Other genes in the vitamin D pathway tested in the current study have been previously associated with asthma or asthma-related phenotypes. In the German Asthma Family Study, Wjst et al. [55] identified SNPs in the *IL10 *gene but also in the *GC*, *CYP2R1 *and *CYP24A1 *genes that were significantly associated with asthma or IgE levels. Interestingly, SNPs in these genes were also associated with serum levels of calcidiol (25-OH-D_3_) and calcitriol [1α,25(OH)_2_D_3_]. In the SLSJ population, three SNPs in the *CYP24A1 *gene showed modest evidence of association with asthma or atopy (p < 0.05). In addition, the only *CYP24A1 *SNP (rs2248359) genotyped in common between the German and SLSJ population showed a consistent result with an increase risk of asthma associated with the C allele. SNPs in the *CYP2R1 *gene, encoding an enzyme implicated in earlier steps of the vitamin D metabolic pathway, were also associated with asthma in both the German and the SLSJ studies. Only one SNP in the *CD86 *and *CD28 *genes reached significance in the SLSJ population. The *CD28 *and *CD86 *interaction represents an important costimulatory signaling pathway for CD4 positive T cell activation and Th2 cytokine production [57]. The association between atopy and the *CD86 *promoter polymorphism rs2715267 was relatively strong in the SLSJ collection (p = 0.004). Different SNPs in the *CD86 *gene were also associated with asthma in the CAPPS and BHS studies. Thus, evidence is building to implicate *CD86 *as a susceptibility gene for asthma, but independent replications in larger population samples are required. In contrast, the overall results for *CD28 *are more consistent with the previous studies showing the lack of association with asthma [58-60]. Many SNPs in the *IL1RL1 *gene were associated with asthma in the SLSJ study. This receptor is required for the development of an effective Th2 response [61,62] and mediates the biological effects of IL-33 [63]. A functional promoter polymorphism (rs6543116) in the *IL1RL1 *gene was previously associated with atopic dermatitis and high total IgE levels in the sera from the same patients [64]. More recently, a genome-wide association scan for blood eosinophil count identified a strong association with an intronic SNP (rs1420101) in this gene [65]. The same SNP was then associated with asthma in a collection of ten different populations (7,996 cases and 44,890 controls). This SNP was not genotyped in the current study, but rs950880 can be used as a surrogate (r^2 ^= 0.96 based on the European HapMap data). rs950880 was not associated with asthma or atopy in any of the four populations evaluated in the current study. However, a trend (p ≤ 0.10) in the same direction as the eosinophil/asthma study [65] was observed for asthma and atopy in the SLSJ study as well as for atopy in the BHS. Finally, the SLSJ results do not support previous observations with the *GC *and *IL8 *genes [55,66,67].

It is reasonable to expect interactions among functionally related genes. In the SLSJ study, some two-gene models were more efficient at evaluating the risk of asthma compared with single SNP models. The models implicating functional variants in the *VDR *and *IL10 *genes are particularly interesting. We previously demonstrated that genetic variants in the *VDR *gene are associated with asthma [21]. Since the latter publication [21], we have genotyped more SNPs in the *VDR *gene in the same population and many SNPs between intron 2 and exon 9 are associated with asthma (see Additional file 12). The mechanism explaining this association is still unclear, but allele-specific expression at this locus implicates the presence of genetic variants that influence *VDR *expression [68]. In fact, we observed that the highly expressed haplotype was overtransmitted to asthmatic individuals, while the low expressed haplotype was undertransmitted [3]. These *VDR *haplotypes can be tagged by the SNP rs1544410 with the G allele being associated with the high-risk/high-expressed haplotype and the A allele being associated with the low-risk/low-expressed haplotype. As mentioned above, the low IL10-producing haplotype was associated with asthma in the same population (SLSJ). These *IL10 *haplotypes can be tagged by the SNP rs1800871 with the T allele being associated with the high-risk/low-producing haplotype and the C allele being associated with the low-risk/high-production haplotype. By combining the information of both SNPs (*IL10*-rs1800871 and *VDR*-rs1544410) we obtained the two-gene model shown in Figure 5b and that was a significant (p = 0.00018) predictor of the risk of asthma. In this model, the risk of asthma increased additively with the number of G alleles (tagging the high-risk/high-expressed haplotype) at the *VDR *locus and with the number of T alleles (tagging the high-risk/low-producing haplotype) at the *IL10 *locus. Similarly, other intriguing interactions between plausible candidate genes were observed in the SLSJ population, including *IL10 *and *IL1RL1 *(Figure 5d) as well as IL10 and CD86 (Figure 5e). Although interesting, the large majority of these two-gene models were not replicated in the other populations. In the CAMP study, a two gene-model involving *IL10 *and *VDR *variants mirrored the observation made in the SLSJ sample. However, further work will be required to demonstrate the relevance of these two-gene models in asthma.

The cascade of enzymatic reactions that lead to the biosynthesis of 1α,25(OH)_2_D_3 _is complex and requires the participation of many gene products (Figure 1). It is conceivable that polymorphisms in any of several genes in this pathway could lead to differences in endogenous biosynthesis and bioavailability of 1α,25(OH)_2_D_3_. At a more downstream step in the pathway, genetic variants in the *VDR *itself or in genes implicated in the transcriptional machinery, can influence the sensitivity of the 1α,25(OH)_2_D_3 _stimulus. Further downstream are transcriptionally regulated genes, or vitamin responsive genes, that mediate the broad physiological actions of 1α,25(OH)_2_D_3_. Again, polymorphisms in these genes are prime candidates to influence the vitamin D response. However, at this level the task is rather challenging owing to the large number of genes regulated by 1α,25(OH)_2_D_3 _[14]. An extra layer of complexity exists, knowing that the regulation of gene expression by 1α,25(OH)_2_D_3 _is highly cell-specific [69,70]. Accordingly, a large number of genes can determine the overall response to the vitamin D pathway. With this in perspective, gene selection in the current study was not comprehensive. On the other hand, the results of the current study may have implications in a wider scope than the field of asthma and asthma-related phenotypes. SNPs associated with asthma in the vitamin D pathway genes may influence the overall vitamin D sensitivity and consequently influence many other diseases [55].

## Conclusion

Cumulative observations implicate the vitamin D pathway in immune responses. By studying genes involved in this pathway, we identified genetic polymorphisms modestly associated with asthma and atopy in asthmatic families from the Saguenay^_^Lac-Saint-Jean region. Significant two-gene models that include interactions between components of the pathway were also identified. We then attempted to replicate the findings in two additional Canadian studies and one Australian study. Similar to the SLSJ study, some SNPs in the *IL10 *and *CYP24A1 *genes were modestly associated with asthma or atopy. However, the SNPs or the orientation of the risk alleles were different between populations. It is conceivable that the effect of the VDR system in immune responses can be overruled by more potent immune pathways. Accordingly, the effect of the vitamin D pathway might be detectable only in specific environments or age-related contexts. Further studies are warranted to confirm the single SNP and the multilocus models associated with asthma in the current study.

## List of abbreviations used

BHS: Busselton Health Study; CAMP: Childhood Asthma Management Program; CAPPS: Canadian Asthma Primary Prevention Study; CD28: CD28 molecule; CD86: CD86 molecule; CYP24A1: cytochrome P450, family 24, subfamily A, polypeptide 1; CYP27A1: cytochrome P450, family 27, subfamily A, polypeptide 1; CYP27B1: cytochrome P450, family 27, subfamily B, polypeptide 1; CYP2R1: cytochrome P450, family 2, subfamily R, polypeptide 1; FBAT: family based association test; FEV_1_: forced expiratory volume in 1 second; GC: vitamin D binding protein; IL10: interleukin 10; IL1RL1: interleukin 1 receptor-like 1; IL8: interleukin 8; LD: linkage disequilibrium; NHS: Nurses' Health Study; PC20: the concentration of methacholine that causes a 20% decline in FEV_1_; SAGE: Study of Asthma Genes and the Environment; SKIIP: SKI interacting protein; SLSJ: Saguenay^_^Lac-Saint-Jean; SNP: single-nucleotide-polymorphism; VDR: vitamin D receptor.

## Competing interests

The authors declare that they have no competing interests.

## Authors' contributions

YB carried out gene/SNP selection, genotyping in the SLSJ study, integration of datasets and was primary author of the manuscript. ML performed statistical analyses in SLSJ, SAGE, CAPPS, and BHS. AHP carried out statistical analyses in CAMP. DD, JQH and AS provided statistical, genotyping and genetics expertise to replicate the findings in the AllerGen study samples. JHW made substantial intellectual contribution in gene selection. ALJ, AWM and LJP participated in the conception and coordination of the BHS. BAR and STW participated in the conception and coordination of CAMP. ALK participated in the coordination of the SAGE study. AB conceived and acquired funding for the SAGE and CAPPS studies. TJH and CL conceived and acquired funding for the SLSJ study. TJH also provided general supervision of the research group. All authors read and approved the final manuscript.

## Supplementary Material

Additional file 1 **SNP characteristics**. Table showing the SNPs selected for genotyping and their characteristics including chromosomal location, type of variation, minor allele, minor allele frequency, and Hardy-Weinberg p value. Click here for file

Additional file 2 **Genetic association of SNPs in the vitamin D pathway genes with asthma and atopy in the SLSJ study**. Each subfigure presents the result of one gene. The top line indicates the gene name and symbol. The upper part of each subfigure shows the exon-intron structure of the gene and the localization of the genotyped SNPs. The coding exons are shown in black and the untranslated regions are shown in grey. The lower part of each subfigure illustrates the association results for asthma (solid circles) and atopy (open circles). The x-axis shows the localization of the gene and SNPs on NCBI Human Genome build 35. The y-axis shows the FBAT empirical p values on a log_10 _scale. The lower and upper dashed lines represent p value thresholds of 0.05 and 0.001, respectively. The upper and lower parts of each subfigure are shown on the same scale. Click here for file

Additional file 3 **The overall distribution of p values derived from single marker FBAT association tests in the SLSJ study**. The top panels are Q-Q plots showing the distribution of observed p values against the expected distribution for asthma and atopy. The bottom panels are histograms showing the distribution of p values for asthma and atopy. The dashed lines represent the mean number of p values that is expected by chance. Association test results for 83 SNPs located in 11 genes are illustrated. Click here for file

Additional file 4 **Single SNP association results for asthma and atopy in the Saguenay^_^Lac-Saint-Jean study**. Table showing the FBAT results for all SNPs in the Saguenay^_^Lac-Saint-Jean study. Click here for file

Additional file 5 **Linkage disequilibrium (LD) plots surrounding eleven genes involved in the vitamin D pathway in the SLSJ study**. The LD plots were generated by Haploview 3.32 [41]. Gene symbols are indicated at the top of each graph. The top horizontal bar illustrates the location of SNPs on a physical scale. The color of squares illustrates the strength of pairwise r^2 ^values on a black and white scale where black indicates perfect LD (r^2 ^= 1.00) and white indicates perfect equilibrium (r^2 ^= 0). The r^2 ^LD value is also indicated within each square. Blocks are defined using the Gabriel et al [71] definition. Failed and monomorphic SNPs as well as SNPs not in Hardy-Weinberg equilibrium are not illustrated. Click here for file

Additional file 6 **Two-gene model analyses on atopy in the SLSJ study**. Visual representation of results is explained in Figure 4. Click here for file

Additional file 7 **Single SNP association results for asthma and atopy in the replication samples**. Table showing the results for all SNPs in the replication samples. Click here for file

Additional file 8 **Two-gene model analyses on asthma in the BHS**. Visual representation of results is explained in Figure 4. Click here for file

Additional file 9 **Single SNP association results for asthma and atopy in combined analyses (SLSJ, CAPPS, SAGE, and BHS)**. Table showing the results for the combined analyses. Click here for file

Additional file 10 **Two-gene model analyses on asthma in the combined dataset for genes involved in the vitamin D pathway**. Visual representation of results is explained in Figure 4. Click here for file

Additional file 11 **Two-gene model analyses on atopy in the combined dataset for genes involved in the vitamin D pathway**. Visual representation of results is explained in Figure 4. Click here for file

Additional file 12 **Genetic association between SNPs in the vitamin D receptor gene and asthma in the SLSJ study**. The upper part of the figure shows the exon-intron structure of the gene and the localization of the genotyped SNPs. The coding exons are shown in black and the untranslated regions are shown in grey. The lower part of the figure illustrates the association results for asthma. The x-axis shows the localization of the gene and SNPs on NCBI Human Genome build 35. The y-axis shows the FBAT empirical p values on a log_10 _scale. The lower and upper dashed lines represent p value thresholds of 0.05 and 0.001, respectively. The upper and lower parts of the figure are shown on the same scale. Click here for file
