# Supplementary material for: Asthma and genes encoding components of the vitamin D pathway
Source: Respir Res. 2009 Oct 24;10(1):98. doi: 10.1186/1465-9921-10-98 (PMC2779188; doi:10.1186/1465-9921-10-98)
Supplement: Additional file 1 — SNP characteristics. Table showing the SNPs selected for genotyping and their characteristics including chromosomal location, type of variation, minor allele, minor allele frequency, and Hardy-Weinberg p value. [file 1465-9921-10-98-S1.DOC]

**Supplementary Table 1**. SNP characteristics.

|  |  |  |  |  |  |  | **SLSJ**^§^ | |
| --- | --- | --- | --- | --- | --- | --- | --- | --- |
| **Gene** | **SNP** | **Location (chr:position)** | **Variation** | **Strand*** | **Minor allele**^†^ | **MAF (%)**  **HapMap**^†^ | **MAF (%)** | **H-W p value** |
| IL10 | rs4844553 | 1:203322758 | C/T | + | T | 6 | 7 | 0.179 |
|  | rs3024505 | 1:203328299 | C/T | - | T | 17 | 17 | 0.060 |
|  | rs3024498 | 1:203329924 | A/G | - | G | 29 | 24 | 0.994 |
|  | rs3024490 | 1:203333706 | G/T | - | T | 22 | 27 | 0.921 |
|  | rs1800872 | 1:203334802 | A/C | - | A | 19 | 27 | 0.938 |
|  | rs1800871 | 1:203335029 | C/T | - | T | 17 | 28 | 0.973 |
|  | rs1800896 | 1:203335292 | A/G | - | A | 46 | 54 | 0.979 |
|  | rs10494879 | 1:203340599 | G/C | + | G | 46 | 39 | 0.846 |
| IL1RL1 | rs950880 | 2:102391080 | G/T | - | T | 32 | 40 | 0.247 |
|  | rs1420089 | 2:102396907 | A/G | - | G | 12 | 17 | 0.001 |
|  | rs1420103 | 2:102407150 | G/T | - | T | 28 | 22 | 0.989 |
|  | **rs1041973** | 2:102413986 | A/C | + | A | 12 | 24 | 0.822 |
|  | rs6719130 | 2:102416754 | C/T | + | T | 19 | 11 | 0.945 |
|  | rs3771175 | 2:102418728 | A/T | + | T | 9 | 13 | 0.613 |
|  | rs1946131 | 2:102420447 | A/G | - | A | 8 | 9 | 0.739 |
|  | rs6543119 | 2:102421590 | A/T | + | T | 36 | 38 | 0.722 |
|  | rs1921622 | 2:102424585 | A/G | + | G | 47 | 52 | 0.963 |
|  | rs1861245 | 2:102425424 | A/G | - | A | 40 | 42 | 0.009 |
|  | **rs4988956** | 2:102426525 | A/G | + | A | 40 | 41 | 0.381 |
|  | **rs10192036** | 2:102426729 | A/C | + | A | NA | 39 | 0.500 |
|  | **rs10204137** | 2:102426730 | A/G | + | G | 40 | 40 | 0.629 |
|  | **rs10192157** | 2:102426874 | C/T | + | T | 40 | 40 | 0.647 |
|  | **rs10206753** | 2:102426880 | C/T | + | C | 40 | 40 | 0.578 |
| CD28 | rs12479446 | 2:204388521 | C/T | + | T | 7 | 12 | 0.088 |
|  | rs1879877 | 2:204395506 | A/C | - | A | 21 | 26 | 0.998 |
|  | rs3181096 | 2:204395598 | C/T | + | T | 37 | 34 | 0.979 |
|  | rs3769683 | 2:204404303 | A/G | + | A | 12 | 13 | 0.613 |
|  | rs3116487 | 2:204411543 | G/C | + | G | 18 | 22 | 0.980 |
|  | rs3116494 | 2:204417527 | A/G | + | G | 27 | 33 | 0.134 |
|  | rs6435203 | 2:204436701 | A/G | + | G | 27 | 33 | 0.807 |
| CYP27A1 | rs4674338 | 2:219472992 | A/G | + | A | 35 | 40 | 0.222 |
|  | rs12623740 | 2:219491220 | A/T | + | A | 41 | 48 | 0.028 |
|  | rs645163 | 2:219507762 | A/G | - | A | 15 | 15 | 0.236 |
|  | rs6436094 | 2:219513102 | A/G | + | G | 23 | 20 | 0.732 |
| CD86 | rs12106790 | 3:123249744 | A/C | + | C | 14 | 22 | 0.600 |
|  | rs2715267 | 3:123253548 | A/C | - | C | 31 | 37 | 0.996 |
|  | rs2715273 | 3:123264641 | A/T | - | T | 18 | 18 | 0.886 |
|  | rs4308217 | 3:123275877 | A/C | + | A | 43 | 27 | 0.970 |
|  | rs9831894 | 3:123283177 | A/C | + | A | 49 | 64 | 0.959 |
|  | rs6805035 | 3:123285838 | A/C | + | C | 16 | 12 | 0.937 |
|  | rs11717893 | 3:123289515 | C/T | + | C | 23 | 26 | 0.650 |
|  | rs2681415 | 3:123298305 | A/G | + | G | 12 | 16 | 0.963 |
|  | rs3792285 | 3:123302276 | A/C | + | A | 8 | 10 | 1.000 |
|  | rs2332096 | 3:123303833 | G/T | + | T | 40 | 46 | 0.951 |
|  | **rs2681417** | 3:123307887 | A/G | + | G | 4 | 6 | 0.003 |
|  | rs9848900 | 3:123314187 | A/G | + | G | 24 | 27 | 0.992 |
|  | **rs1129055** | 3:123321009 | A/G | + | A | 28 | 29 | 0.998 |
|  | rs1915087 | 3:123321481 | C/T | + | C | 31 | failed | failed |
|  | rs2681401 | 3:123325817 | G/T | + | T | 48 | 36 | 0.571 |
| GC | rs1491711 | 4:72967287 | G/C | - | C | 37 | failed | failed |
|  | rs705117 | 4:72973150 | A/G | - | G | 13 | 12 | 0.903 |
|  | rs1491709 | 4:72978601 | C/T | - | T | 9 | 3 | 0.822 |
|  | **rs4588** | 4:72983358 | A/C | - | A | NA | 34 | 0.324 |
|  | **rs7041** | 4:72983369 | G/T | - | T | 43 | 46 | 0.984 |
|  | rs403376 | 4:72993921 | A/T | + | A | 0 | 11 | 0.709 |
|  | rs222014 | 4:72997966 | A/G | - | A | 11 | 9 | 0.808 |
|  | rs222029 | 4:73009997 | A/G | + | G | 17 | 14 | 0.517 |
|  | rs2298849 | 4:73013886 | C/T | - | C | 21 | 19 | 0.011 |
| IL8 | rs4073 | 4:74971059 | A/T | + | A | NA | 49 | 0.923 |
|  | rs2227306 | 4:74972090 | C/T | + | T | 39 | 46 | 0.354 |
|  | rs1126647 | 4:74974080 | A/T | + | T | NA | 47 | 0.670 |
|  | rs16849958 | 4:74982070 | A/C | + | C | 41 | 47 | 0.506 |
| CYP2R1 | rs11023371 | 11:14852847 | A/G | + | A | 8 | 8 | 0.341 |
|  | rs11023374 | 11:14860212 | C/T | + | C | 27 | 32 | 0.864 |
|  | rs7936142 | 11:14866321 | A/T | + | T | 10 | 13 | 0.754 |
|  | rs1993116 | 11:14866810 | C/T | - | T | 37 | 36 | 0.759 |
|  | rs10500804 | 11:14866849 | G/T | + | G | 43 | 43 | 0.949 |
|  | rs1562902 | 11:14874792 | C/T | + | C | 42 | 51 | 0.869 |
| CYP27B1 | rs1048691 | 12:56439215 | C/T | + | T | 22 | 25 | 0.013 |
|  | rs8176353 | 12:56441489 | A/T | - | T | 0 | 0 | NA |
|  | rs4646536 | 12:56444255 | C/T | - | C | NA | 33 | 0.571 |
|  | rs8176341 | 12:56448165 | C/G | + | G | NA | 24 | 0.013 |
| SKIIP | rs176965 | 14:77257069 | A/C | + | A | 40 | 37 | 0.984 |
|  | rs1030151 | 14:77275608 | C/T | + | T | 8 | 17 | 0.001 |
|  | rs4346144 | 14:77288897 | A/G | + | A | 12 | 14 | 0.105 |
|  | rs1477261 | 14:77290731 | A/T | + | A | 14 | 18 | 0.918 |
|  | rs11621593 | 14:77293958 | C/T | + | T | 22 | 16 | 0.340 |
|  | rs2277917 | 14:77297385 | G/C | + | C | 48 | 46 | 0.727 |
|  | rs11628795 | 14:77304965 | G/T | + | T | 26 | 21 | 0.826 |
| CYP24A1 | rs8124792 | 20:52200214 | A/G | + | A | 8 | 5 | 0.987 |
|  | rs6097801 | 20:52200841 | A/G | + | A | 8 | 16 | 0.674 |
|  | rs927650 | 20:52206148 | C/T | + | C | 43 | 55 | 0.264 |
|  | rs912505 | 20:52210248 | A/G | + | G | 15 | 29 | 0.021 |
|  | rs6068816 | 20:52214498 | C/T | + | T | 12 | 14 | 0.346 |
|  | rs4809960 | 20:52219480 | C/T | + | C | 22 | 29 | 0.000 |
|  | rs2248359 | 20:52224925 | C/T | + | T | 38 | 43 | 0.937 |
|  | rs2426498 | 20:52230088 | G/C | + | G | 13 | 9 | 0.439 |

SLSJ, Saguenay−Lac-Saint-Jean; H-W, Hardy-Weinberg; MAF, minor allele frequency; NA, not available.

Coding non-synonymous SNPs are shown in bold.

^*^Strand relative to the human reference sequence.

^†^Based on the CEPH HapMap population (Utah residents with ancestry from northern and western Europe). If not available, the minor allele was determined based on the SLSJ study.

^§^Only parents were considered (n = 402).
