# Supplementary figures and images for: Asthma and genes encoding components of the vitamin D pathway
Source: Respir Res. 2009 Oct 24;10(1):98. doi: 10.1186/1465-9921-10-98 (PMC2779188; doi:10.1186/1465-9921-10-98)

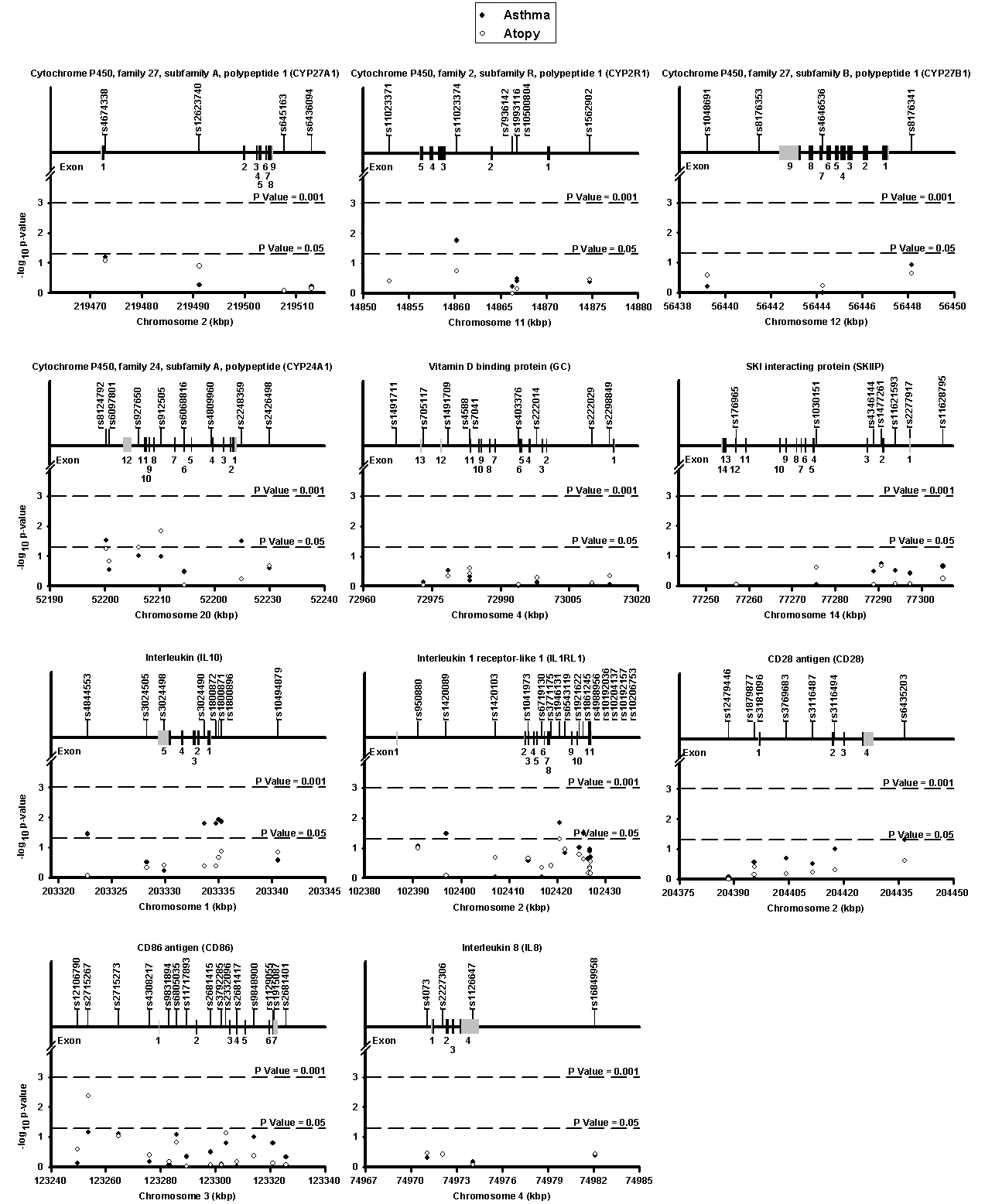

Supplement: Additional file 2 — Genetic association of SNPs in the vitamin D pathway genes with asthma and atopy in the SLSJ study. Each subfigure presents the result of one gene. The top line indicates the gene name and symbol. The upper part of each subfigure shows the exon-intron structure of the gene and the localization of the genotyped SNPs. The coding exons are shown in black and the untranslated regions are shown in grey. The lower part of each subfigure illustrates the association results for asthma (solid circles) and atopy (open circles). The x-axis shows the localization of the gene and SNPs on NCBI Human Genome build 35. The y-axis shows the FBAT empirical p values on a log10 scale. The lower and upper dashed lines represent p value thresholds of 0.05 and 0.001, respectively. The upper and lower parts of each subfigure are shown on the same scale. [file 1465-9921-10-98-S2.JPEG]

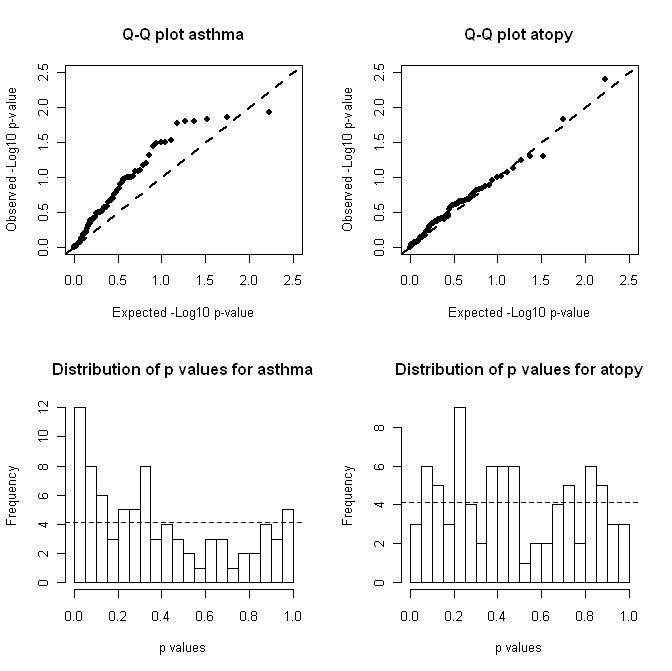

Supplement: Additional file 3 — The overall distribution of p values derived from single marker FBAT association tests in the SLSJ study. The top panels are Q-Q plots showing the distribution of observed p values against the expected distribution for asthma and atopy. The bottom panels are histograms showing the distribution of p values for asthma and atopy. The dashed lines represent the mean number of p values that is expected by chance. Association test results for 83 SNPs located in 11 genes are illustrated. [file 1465-9921-10-98-S3.JPEG]

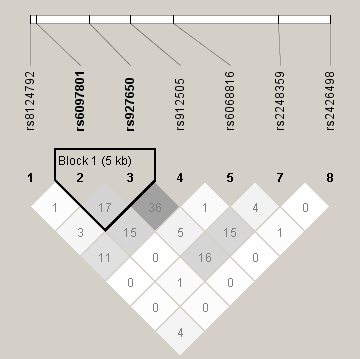


**CYP24A1**


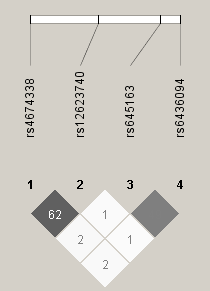


**CYP27A1**


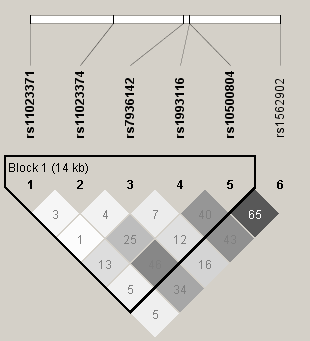


**CYP2R1**


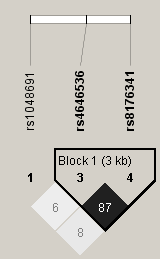


**CYP27B1**


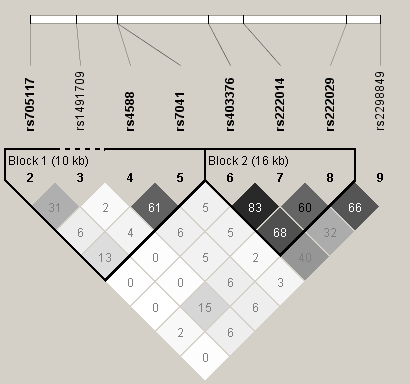


**GC**


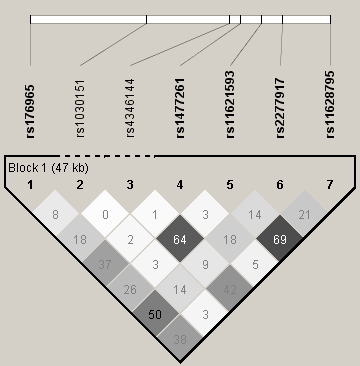


**SKIIP**


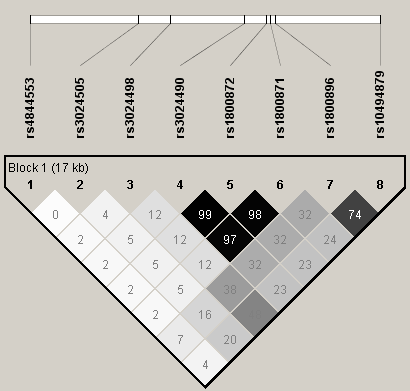


**IL10**


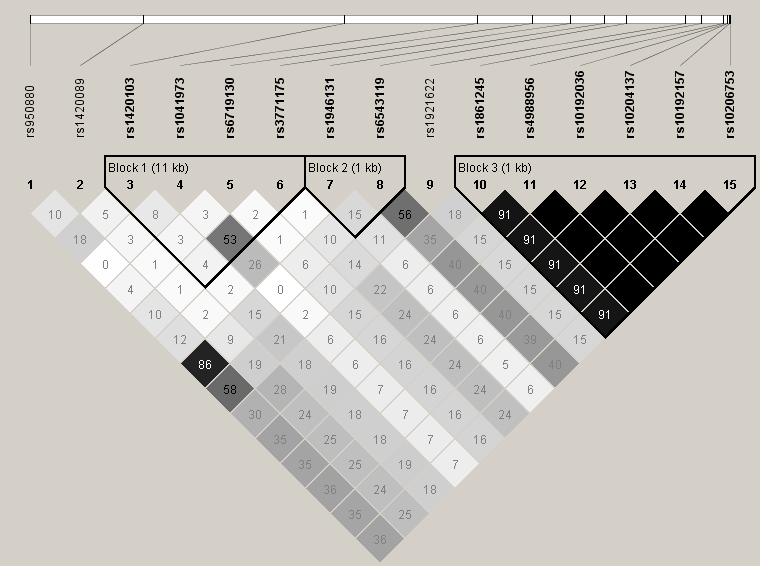


**IL1RL1**


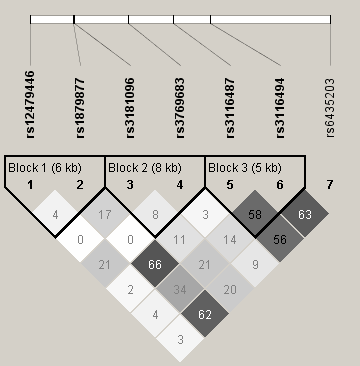


**CD28**


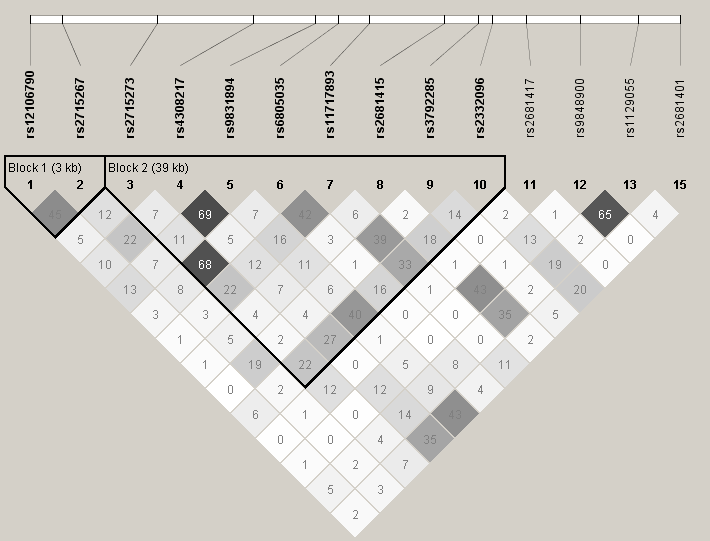


**CD86**


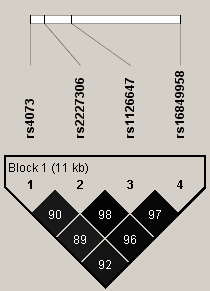


**IL8**

Supplement: Additional file 5 — Linkage disequilibrium (LD) plots surrounding eleven genes involved in the vitamin D pathway in the SLSJ study. The LD plots were generated by Haploview 3.32 [41]. Gene symbols are indicated at the top of each graph. The top horizontal bar illustrates the location of SNPs on a physical scale. The color of squares illustrates the strength of pairwise r2 values on a black and white scale where black indicates perfect LD (r2 = 1.00) and white indicates perfect equilibrium (r2 = 0). The r2 LD value is also indicated within each square. Blocks are defined using the Gabriel et al [71] definition. Failed and monomorphic SNPs as well as SNPs not in Hardy-Weinberg equilibrium are not illustrated. [file 1465-9921-10-98-S5.DOC]

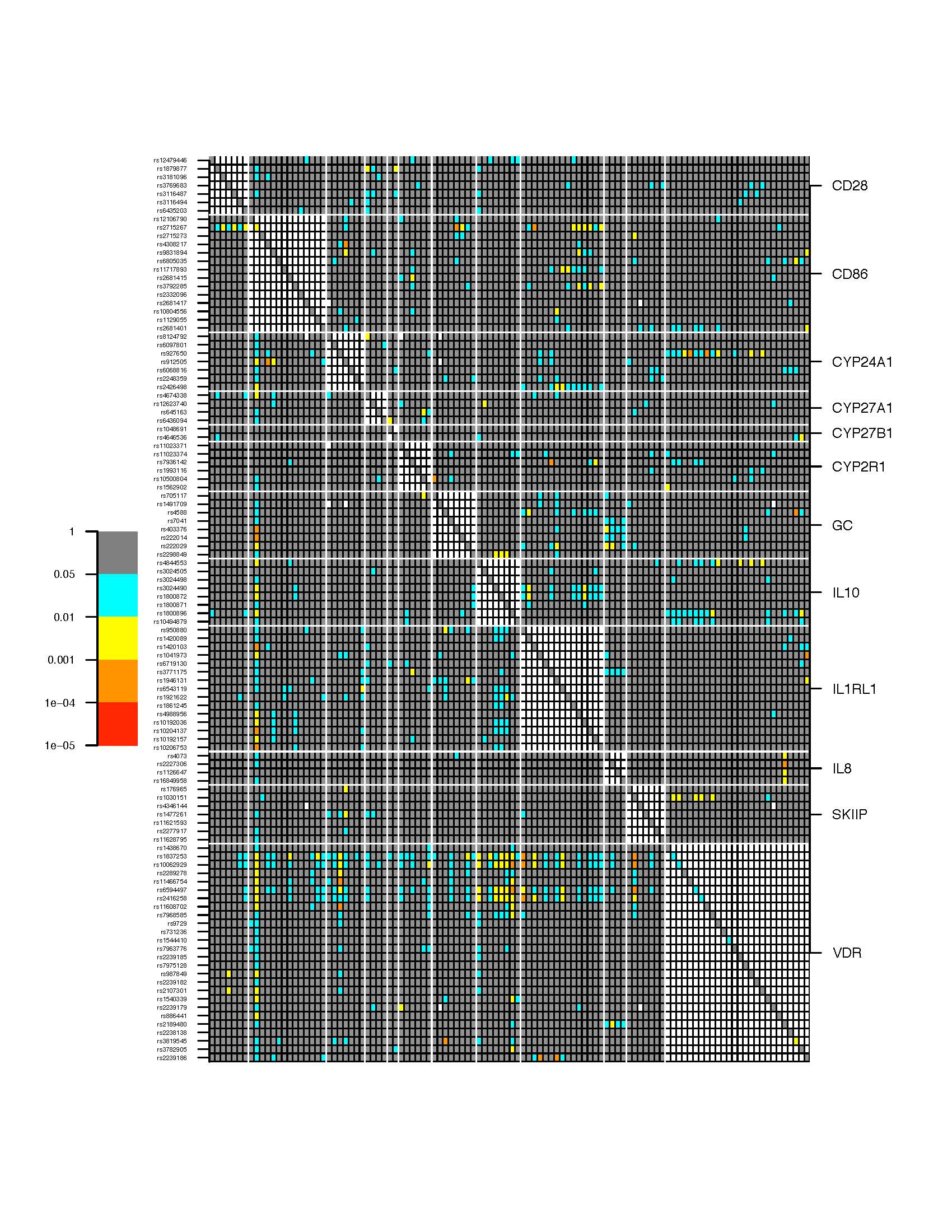

Supplement: Additional file 6 — Two-gene model analyses on atopy in the SLSJ study. Visual representation of results is explained in Figure 4. [file 1465-9921-10-98-S6.JPEG]

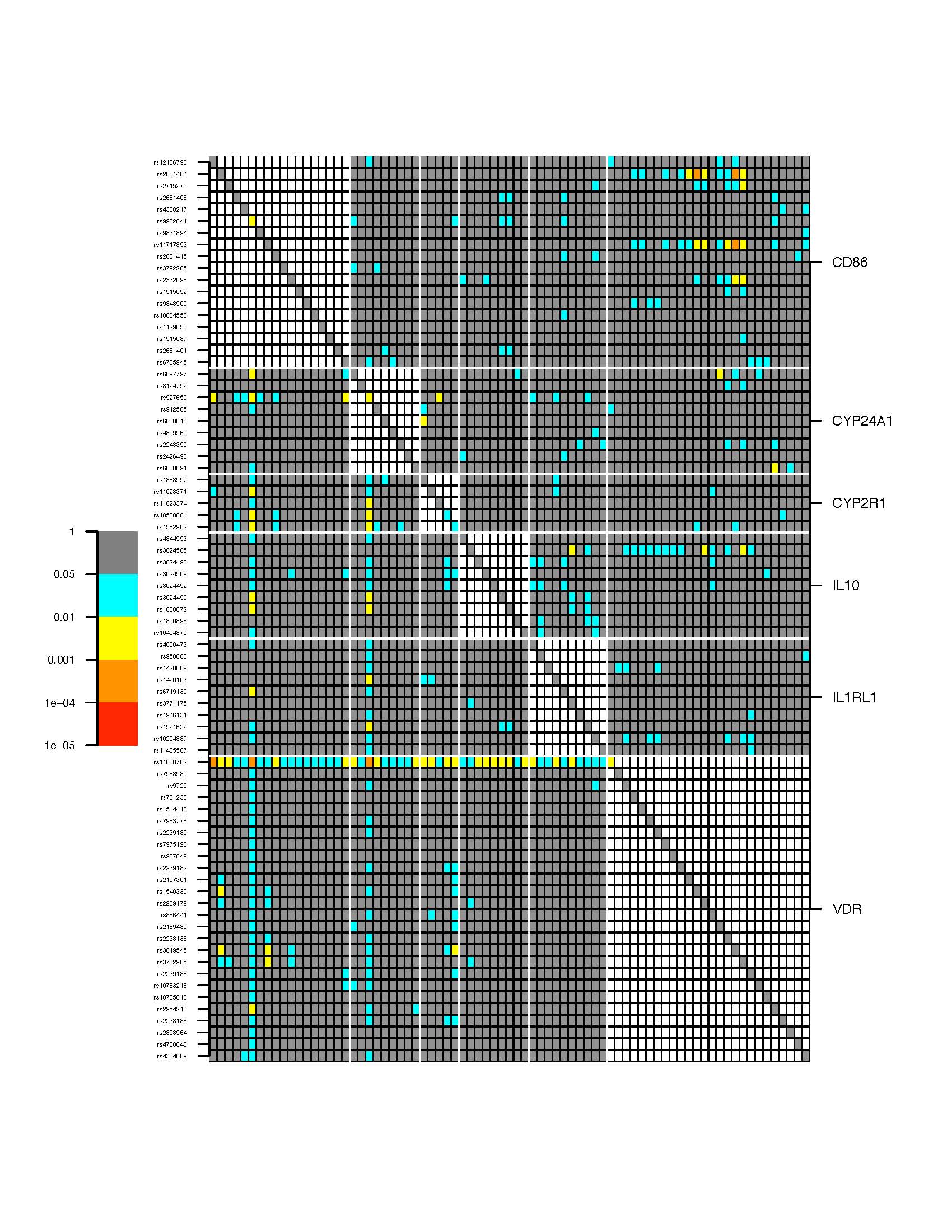

Supplement: Additional file 8 — Two-gene model analyses on asthma in the BHS. Visual representation of results is explained in Figure 4. [file 1465-9921-10-98-S8.JPEG]

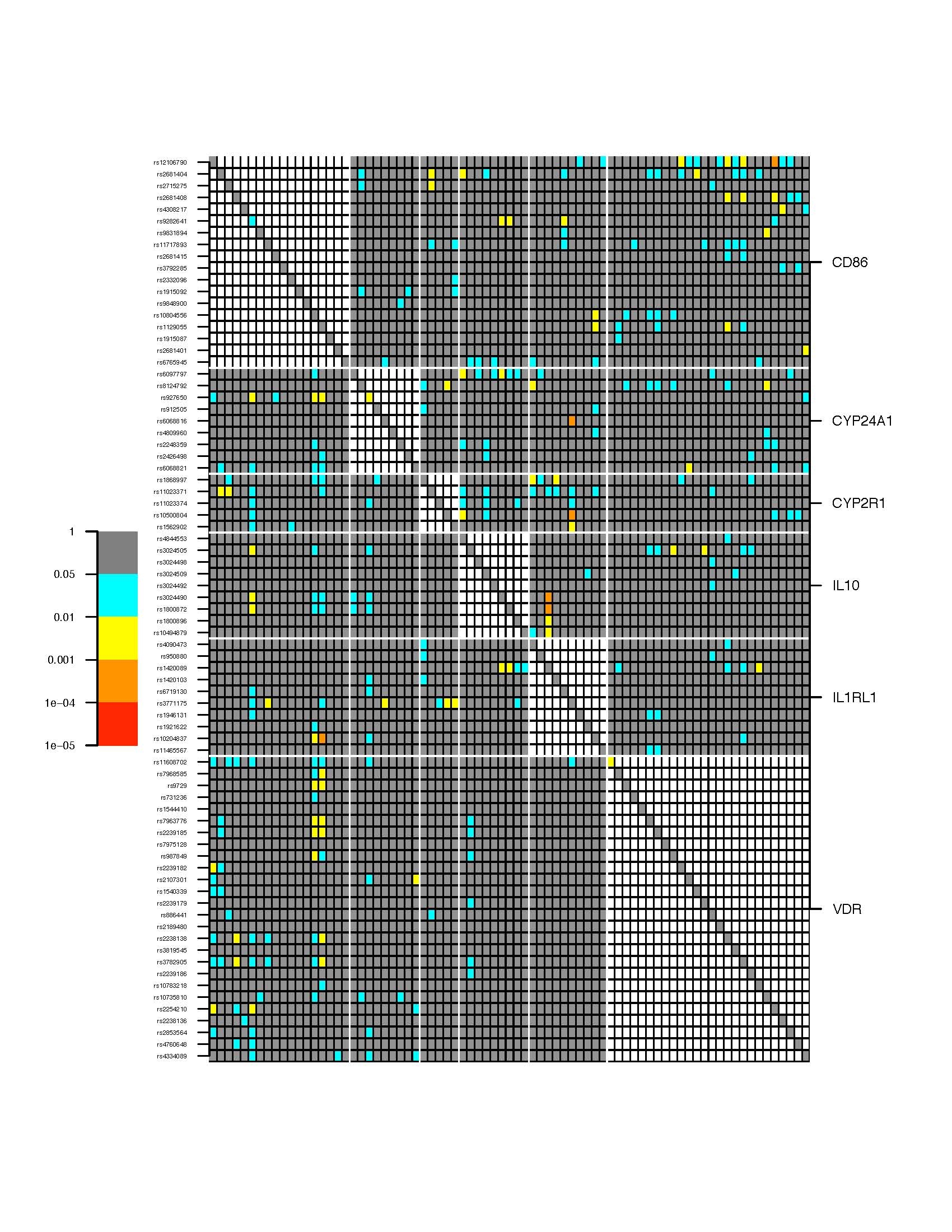

Supplement: Additional file 10 — Two-gene model analyses on asthma in the combined dataset for genes involved in the vitamin D pathway. Visual representation of results is explained in Figure 4. [file 1465-9921-10-98-S10.JPEG]

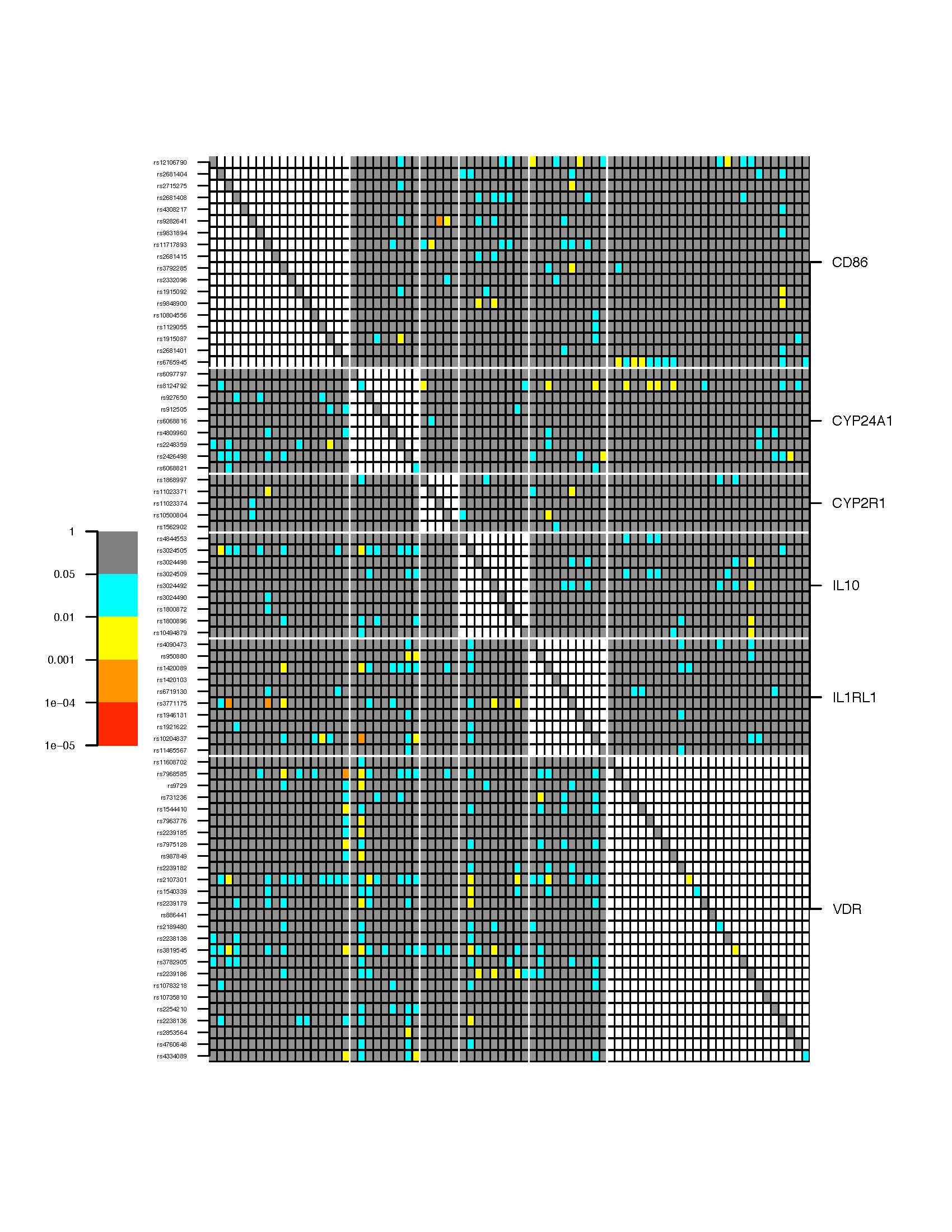

Supplement: Additional file 11 — Two-gene model analyses on atopy in the combined dataset for genes involved in the vitamin D pathway. Visual representation of results is explained in Figure 4. [file 1465-9921-10-98-S11.JPEG]

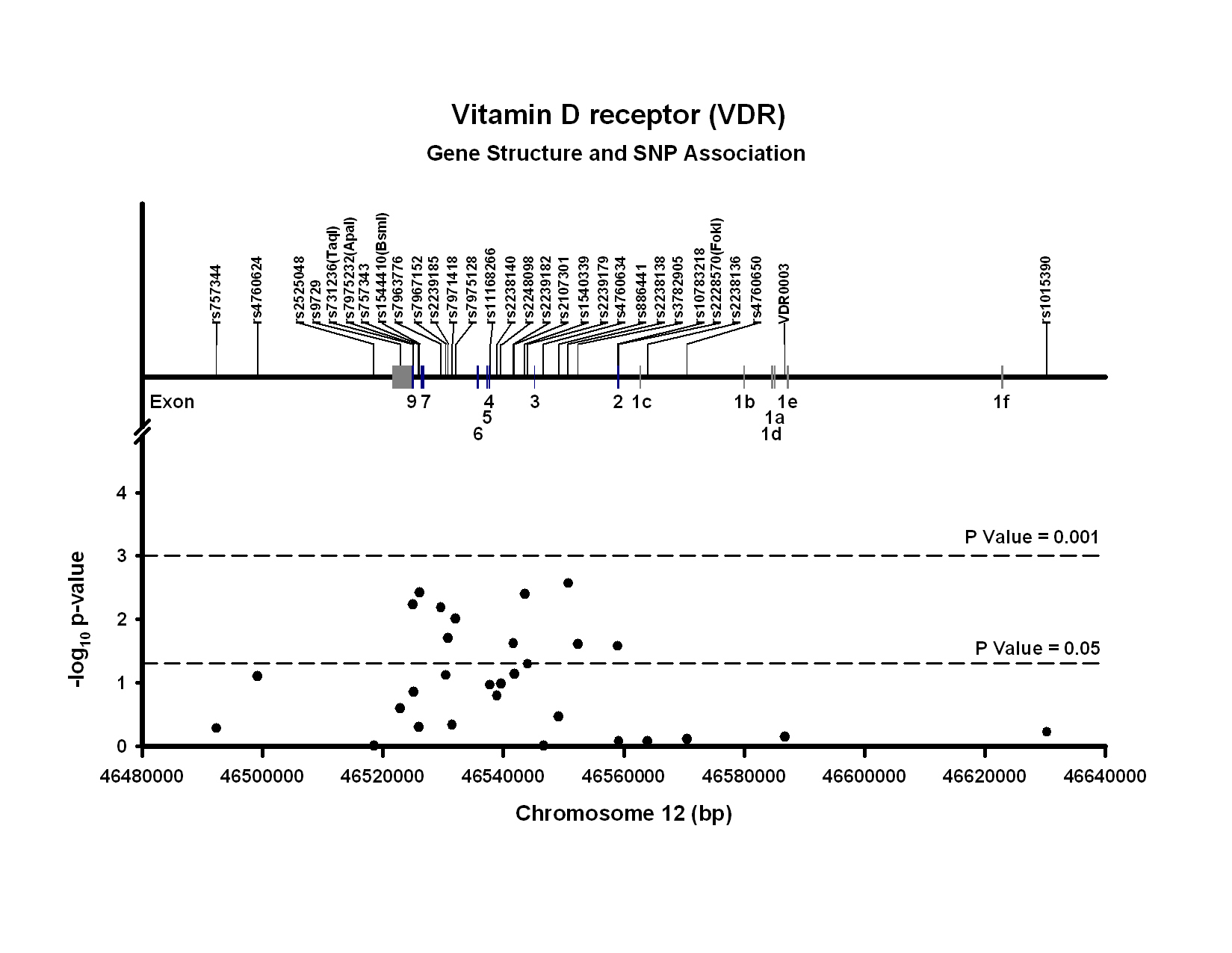

Supplement: Additional file 12 — Genetic association between SNPs in the vitamin D receptor gene and asthma in the SLSJ study. The upper part of the figure shows the exon-intron structure of the gene and the localization of the genotyped SNPs. The coding exons are shown in black and the untranslated regions are shown in grey. The lower part of the figure illustrates the association results for asthma. The x-axis shows the localization of the gene and SNPs on NCBI Human Genome build 35. The y-axis shows the FBAT empirical p values on a log10 scale. The lower and upper dashed lines represent p value thresholds of 0.05 and 0.001, respectively. The upper and lower parts of the figure are shown on the same scale. [file 1465-9921-10-98-S12.JPEG]
