# Supplementary material for: Asthma and genes encoding components of the vitamin D pathway
Source: Respir Res. 2009 Oct 24;10(1):98. doi: 10.1186/1465-9921-10-98 (PMC2779188; doi:10.1186/1465-9921-10-98)
Supplement: Additional file 4 — Single SNP association results for asthma and atopy in the Saguenay_Lac-Saint-Jean study. Table showing the FBAT results for all SNPs in the Saguenay_Lac-Saint-Jean study. [file 1465-9921-10-98-S4.DOC]

**Supplementary Table 2**. Single SNP association results for asthma and atopy in the Saguenay-Lac-Saint-Jean study.

|  |  | **asthma** | | | | |  | **atopy** | | | |
| --- | --- | --- | --- | --- | --- | --- | --- | --- | --- | --- | --- |
|  |  | **Allele** | **Allele frequency** | **# of families*** | **Z** | **P value** |  | **Allele frequency** | **# of families*** | **Z** | **P value** |
| IL10 | rs4844553 | C | 0.94 | 39 | 2.10 | **0.036** |  | 0.94 | 33 | 0.15 | 0.881 |
|  |  | T | 0.06 | 39 | -2.10 | **0.036** |  | 0.06 | 33 | -0.15 | 0.881 |
|  | rs3024505 | A | 0.18 | 65 | -1.00 | 0.316 |  | 0.18 | 53 | -0.71 | 0.479 |
|  |  | G | 0.82 | 65 | 1.00 | 0.316 |  | 0.82 | 53 | 0.71 | 0.479 |
|  | rs3024498 | C | 0.24 | 97 | -0.51 | 0.613 |  | 0.24 | 82 | -0.85 | 0.394 |
|  |  | T | 0.76 | 97 | 0.51 | 0.613 |  | 0.76 | 82 | 0.85 | 0.394 |
|  | rs3024490 | A | 0.28 | 105 | 2.42 | **0.016** |  | 0.28 | 94 | 0.80 | 0.423 |
|  |  | C | 0.72 | 105 | -2.42 | **0.016** |  | 0.72 | 94 | -0.80 | 0.423 |
|  | rs1800872 | A | 0.28 | 105 | 2.42 | **0.016** |  | 0.28 | 94 | 0.80 | 0.423 |
|  |  | C | 0.72 | 105 | -2.42 | **0.016** |  | 0.72 | 94 | -0.80 | 0.423 |
|  | rs1800871 | C | 0.71 | 95 | -2.52 | **0.012** |  | 0.71 | 84 | -1.22 | 0.221 |
|  |  | T | 0.29 | 95 | 2.52 | **0.012** |  | 0.29 | 84 | 1.22 | 0.221 |
|  | rs1800896 | C | 0.46 | 117 | -2.47 | **0.014** |  | 0.46 | 101 | -1.49 | 0.137 |
|  |  | T | 0.54 | 117 | 2.47 | **0.014** |  | 0.54 | 101 | 1.49 | 0.137 |
|  | rs10494879 | C | 0.61 | 115 | 1.11 | 0.269 |  | 0.61 | 101 | 1.47 | 0.143 |
|  |  | G | 0.40 | 115 | -1.11 | 0.269 |  | 0.40 | 101 | -1.47 | 0.143 |
| IL1RL1 | rs950880 | A | 0.39 | 126 | 1.73 | 0.084 |  | 0.39 | 105 | 1.64 | 0.100 |
|  |  | C | 0.61 | 126 | -1.73 | 0.084 |  | 0.61 | 105 | -1.64 | 0.100 |
|  | rs1420089 | C | 0.18 | 72 | -2.14 | **0.033** |  | 0.18 | 60 | -0.21 | 0.832 |
|  |  | T | 0.82 | 72 | 2.14 | **0.033** |  | 0.82 | 60 | 0.21 | 0.832 |
|  | rs1420103 | A | 0.23 | 98 | -0.07 | 0.944 |  | 0.23 | 90 | -1.25 | 0.211 |
|  |  | C | 0.77 | 98 | 0.07 | 0.944 |  | 0.77 | 90 | 1.25 | 0.211 |
|  | rs1041973 | A | 0.23 | 102 | 1.10 | 0.271 |  | 0.23 | 86 | 1.22 | 0.221 |
|  |  | C | 0.77 | 102 | -1.10 | 0.271 |  | 0.77 | 86 | -1.22 | 0.221 |
|  | rs6719130 | C | 0.88 | 59 | 0.11 | 0.915 |  | 0.88 | 56 | 0.74 | 0.458 |
|  |  | T | 0.12 | 59 | -0.11 | 0.915 |  | 0.12 | 56 | -0.74 | 0.458 |
|  | rs3771175 | A | 0.86 | 72 | 0.82 | 0.413 |  | 0.86 | 61 | 0.86 | 0.392 |
|  |  | T | 0.14 | 72 | -0.82 | 0.413 |  | 0.14 | 61 | -0.86 | 0.392 |
|  | rs1946131 | C | 0.92 | 53 | -2.44 | **0.015** |  | 0.92 | 42 | -1.96 | **0.050** |
|  |  | T | 0.09 | 53 | 2.44 | **0.015** |  | 0.09 | 42 | 1.96 | **0.050** |
|  | rs6543119 | A | 0.63 | 122 | -1.45 | 0.147 |  | 0.63 | 101 | -1.59 | 0.112 |
|  |  | T | 0.37 | 122 | 1.45 | 0.147 |  | 0.37 | 101 | 1.59 | 0.112 |
|  | rs1921622 | C | 0.54 | 75 | -1.65 | 0.099 |  | 0.54 | 65 | -1.39 | 0.165 |
|  |  | T | 0.46 | 75 | 1.65 | 0.099 |  | 0.46 | 65 | 1.39 | 0.165 |
|  | rs1861245 | A | 0.43 | 101 | -2.14 | **0.032** |  | 0.43 | 74 | -1.19 | 0.235 |
|  |  | G | 0.57 | 101 | 2.14 | **0.032** |  | 0.57 | 74 | 1.19 | 0.235 |
|  | rs4988956 | C | 0.59 | 102 | 1.20 | 0.229 |  | 0.59 | 79 | 0.41 | 0.683 |
|  |  | T | 0.41 | 102 | -1.20 | 0.229 |  | 0.41 | 79 | -0.41 | 0.683 |
|  | rs10192036 | A | 0.41 | 115 | -1.61 | 0.108 |  | 0.41 | 90 | -0.77 | 0.440 |
|  |  | C | 0.60 | 115 | 1.61 | 0.108 |  | 0.60 | 90 | 0.77 | 0.440 |
|  | rs10204137 | C | 0.41 | 115 | -1.53 | 0.127 |  | 0.41 | 90 | -0.69 | 0.488 |
|  |  | T | 0.59 | 115 | 1.53 | 0.127 |  | 0.59 | 90 | 0.69 | 0.488 |
|  | rs10192157 | C | 0.59 | 103 | 1.28 | 0.201 |  | 0.59 | 78 | 1.07 | 0.285 |
|  |  | T | 0.41 | 103 | -1.28 | 0.201 |  | 0.41 | 78 | -1.07 | 0.285 |
|  | rs10206753 | C | 0.41 | 114 | -1.25 | 0.212 |  | 0.41 | 88 | -0.37 | 0.710 |
|  |  | T | 0.59 | 114 | 1.25 | 0.212 |  | 0.59 | 88 | 0.37 | 0.710 |
| CD28 | rs12479446 | A | 0.13 | 51 | 0.19 | 0.846 |  | 0.13 | 40 | 0.00 | 1.000 |
|  |  | G | 0.87 | 51 | -0.19 | 0.846 |  | 0.87 | 40 | 0.00 | 1.000 |
|  | rs1879877 | A | 0.26 | 104 | -1.09 | 0.274 |  | 0.26 | 91 | -0.35 | 0.728 |
|  |  | C | 0.74 | 104 | 1.09 | 0.274 |  | 0.74 | 91 | 0.35 | 0.728 |
|  | rs3181096 | C | 0.67 | 98 | -0.02 | 0.983 |  | 0.67 | 89 | 0.83 | 0.405 |
|  |  | T | 0.33 | 98 | 0.02 | 0.983 |  | 0.33 | 89 | -0.83 | 0.405 |
|  | rs3769683 | C | 0.87 | 73 | 1.27 | 0.203 |  | 0.87 | 58 | 0.42 | 0.671 |
|  |  | T | 0.14 | 73 | -1.27 | 0.203 |  | 0.14 | 58 | -0.42 | 0.671 |
|  | rs3116487 | C | 0.22 | 94 | -1.00 | 0.316 |  | 0.22 | 85 | -0.52 | 0.602 |
|  |  | G | 0.78 | 94 | 1.00 | 0.316 |  | 0.78 | 85 | 0.52 | 0.602 |
|  | rs3116494 | C | 0.33 | 114 | -1.64 | 0.101 |  | 0.33 | 102 | -0.68 | 0.500 |
|  |  | T | 0.67 | 114 | 1.64 | 0.101 |  | 0.67 | 102 | 0.68 | 0.500 |
|  | rs6435203 | C | 0.34 | 120 | -1.97 | **0.049** |  | 0.34 | 104 | -1.17 | 0.243 |
|  |  | T | 0.67 | 120 | 1.97 | **0.049** |  | 0.67 | 104 | 1.17 | 0.243 |
| CYP27A1 | rs4674338 | C | 0.60 | 96 | 1.85 | 0.064 |  | 0.60 | 82 | 1.72 | 0.085 |
|  |  | T | 0.41 | 96 | -1.85 | 0.064 |  | 0.41 | 82 | -1.72 | 0.085 |
|  | rs12623740 | A | 0.51 | 102 | 0.60 | 0.547 |  | 0.51 | 91 | 1.52 | 0.129 |
|  |  | T | 0.49 | 102 | -0.60 | 0.547 |  | 0.49 | 91 | -1.52 | 0.129 |
|  | rs645163 | C | 0.86 | 78 | -0.14 | 0.889 |  | 0.86 | 70 | 0.18 | 0.856 |
|  |  | T | 0.14 | 78 | 0.14 | 0.889 |  | 0.14 | 70 | -0.18 | 0.856 |
|  | rs6436094 | A | 0.81 | 93 | 0.50 | 0.620 |  | 0.81 | 79 | 0.35 | 0.727 |
|  |  | G | 0.19 | 93 | -0.50 | 0.620 |  | 0.19 | 79 | -0.35 | 0.727 |
| CD86 | rs12106790 | A | 0.79 | 83 | -0.31 | 0.755 |  | 0.79 | 70 | -1.13 | 0.257 |
|  |  | C | 0.21 | 83 | 0.31 | 0.755 |  | 0.21 | 70 | 1.13 | 0.257 |
|  | rs2715267 | A | 0.64 | 102 | -1.82 | 0.069 |  | 0.64 | 91 | -2.86 | **0.004** |
|  |  | C | 0.37 | 102 | 1.82 | 0.069 |  | 0.37 | 91 | 2.86 | **0.004** |
|  | rs2715273 | A | 0.82 | 79 | 1.75 | 0.081 |  | 0.82 | 65 | 1.67 | 0.096 |
|  |  | T | 0.18 | 79 | -1.75 | 0.081 |  | 0.18 | 65 | -1.67 | 0.096 |
|  | rs4308217 | A | 0.28 | 105 | -0.44 | 0.662 |  | 0.28 | 91 | -0.83 | 0.404 |
|  |  | C | 0.72 | 105 | 0.44 | 0.662 |  | 0.72 | 91 | 0.83 | 0.404 |
|  | rs9831894 | A | 0.64 | 86 | -0.17 | 0.864 |  | 0.64 | 88 | 0.42 | 0.671 |
|  |  | C | 0.37 | 86 | 0.17 | 0.864 |  | 0.37 | 88 | -0.42 | 0.671 |
|  | rs6805035 | A | 0.88 | 51 | 1.74 | 0.083 |  | 0.88 | 43 | 1.43 | 0.154 |
|  |  | C | 0.12 | 51 | -1.74 | 0.083 |  | 0.12 | 43 | -1.43 | 0.154 |
|  | rs11717893 | C | 0.26 | 81 | -0.75 | 0.454 |  | 0.26 | 70 | -0.04 | 0.970 |
|  |  | T | 0.74 | 81 | 0.75 | 0.454 |  | 0.74 | 70 | 0.04 | 0.970 |
|  | rs2681415 | A | 0.84 | 65 | -1.00 | 0.317 |  | 0.84 | 50 | -0.21 | 0.831 |
|  |  | G | 0.16 | 65 | 1.00 | 0.317 |  | 0.16 | 50 | 0.21 | 0.831 |
|  | rs3792285 | A | 0.11 | 67 | -0.23 | 0.820 |  | 0.11 | 59 | 0.20 | 0.844 |
|  |  | C | 0.90 | 67 | 0.23 | 0.820 |  | 0.90 | 59 | -0.20 | 0.844 |
|  | rs2332096 | A | 0.46 | 117 | -1.39 | 0.164 |  | 0.46 | 108 | -1.79 | 0.074 |
|  |  | C | 0.54 | 117 | 1.39 | 0.164 |  | 0.54 | 108 | 1.79 | 0.074 |
|  | rs2681417 | C | 0.06 | 31 | -0.02 | 0.982 |  | 0.06 | 27 | -0.41 | 0.684 |
|  |  | T | 0.94 | 31 | 0.02 | 0.982 |  | 0.94 | 27 | 0.41 | 0.684 |
|  | rs10804556 | C | 0.30 | 90 | 1.64 | 0.100 |  | 0.30 | 82 | 0.80 | 0.425 |
|  |  | T | 0.70 | 90 | -1.64 | 0.100 |  | 0.70 | 82 | -0.80 | 0.425 |
|  | rs1129055 | C | 0.70 | 102 | -1.41 | 0.157 |  | 0.70 | 88 | -0.30 | 0.768 |
|  |  | T | 0.30 | 102 | 1.41 | 0.157 |  | 0.30 | 88 | 0.30 | 0.768 |
|  | rs2681401 | A | 0.36 | 108 | 0.73 | 0.464 |  | 0.36 | 100 | 0.18 | 0.856 |
|  |  | C | 0.64 | 108 | -0.73 | 0.464 |  | 0.64 | 100 | -0.18 | 0.856 |
| GC | rs705117 | A | 0.89 | 62 | 0.33 | 0.741 |  | 0.89 | 55 | -0.09 | 0.927 |
|  |  | G | 0.11 | 62 | -0.33 | 0.741 |  | 0.11 | 55 | 0.09 | 0.927 |
|  | rs1491709 | C | 0.96 | 17 | 1.03 | 0.306 |  | 0.96 | 17 | 0.74 | 0.461 |
|  |  | T | 0.04 | 17 | -1.03 | 0.306 |  | 0.04 | 17 | -0.74 | 0.461 |
|  | rs4588 | A | 0.35 | 118 | 0.68 | 0.496 |  | 0.35 | 106 | 1.14 | 0.256 |
|  |  | C | 0.65 | 118 | -0.68 | 0.496 |  | 0.65 | 106 | -1.14 | 0.256 |
|  | rs7041 | A | 0.46 | 117 | 0.45 | 0.656 |  | 0.46 | 105 | 0.87 | 0.384 |
|  |  | C | 0.54 | 117 | -0.45 | 0.656 |  | 0.54 | 105 | -0.87 | 0.384 |
|  | rs403376 | A | 0.91 | 53 | -0.04 | 0.970 |  | 0.91 | 49 | -0.14 | 0.892 |
|  |  | T | 0.09 | 53 | 0.04 | 0.970 |  | 0.09 | 49 | 0.14 | 0.892 |
|  | rs222014 | C | 0.92 | 43 | -0.31 | 0.758 |  | 0.92 | 40 | -0.63 | 0.531 |
|  |  | T | 0.08 | 43 | 0.31 | 0.758 |  | 0.08 | 40 | 0.63 | 0.531 |
|  | rs222029 | C | 0.13 | 48 | -0.13 | 0.898 |  | 0.13 | 45 | 0.30 | 0.767 |
|  |  | T | 0.87 | 48 | 0.13 | 0.898 |  | 0.87 | 45 | -0.30 | 0.767 |
|  | rs2298849 | C | 0.19 | 65 | 0.17 | 0.868 |  | 0.19 | 58 | 0.75 | 0.452 |
|  |  | T | 0.82 | 65 | -0.17 | 0.868 |  | 0.82 | 58 | -0.75 | 0.452 |
| IL8 | rs4073 | A | 0.48 | 111 | -0.66 | 0.507 |  | 0.48 | 99 | -0.94 | 0.346 |
|  |  | T | 0.52 | 111 | 0.66 | 0.507 |  | 0.52 | 99 | 0.94 | 0.346 |
|  | rs2227306 | C | 0.55 | 118 | 0.87 | 0.383 |  | 0.55 | 100 | 0.88 | 0.379 |
|  |  | T | 0.45 | 118 | -0.87 | 0.383 |  | 0.45 | 100 | -0.88 | 0.379 |
|  | rs1126647 | A | 0.54 | 111 | 0.40 | 0.688 |  | 0.54 | 94 | 0.19 | 0.850 |
|  |  | T | 0.46 | 111 | -0.40 | 0.688 |  | 0.46 | 94 | -0.19 | 0.850 |
|  | rs16849958 | A | 0.54 | 119 | 0.83 | 0.406 |  | 0.54 | 104 | 0.89 | 0.375 |
|  |  | C | 0.46 | 119 | -0.83 | 0.406 |  | 0.46 | 104 | -0.89 | 0.375 |
| CYP2R1 | rs11023371 | C | 0.94 | 35 | 0.86 | 0.390 |  | 0.94 | 36 | 0.84 | 0.400 |
|  |  | T | 0.06 | 35 | -0.86 | 0.390 |  | 0.06 | 36 | -0.84 | 0.400 |
|  | rs11023374 | C | 0.33 | 105 | 2.38 | **0.017** |  | 0.33 | 88 | 1.33 | 0.183 |
|  |  | T | 0.67 | 105 | -2.38 | **0.017** |  | 0.67 | 88 | -1.33 | 0.183 |
|  | rs7936142 | A | 0.86 | 66 | 0.53 | 0.599 |  | 0.86 | 55 | 0.00 | 1.000 |
|  |  | T | 0.14 | 66 | -0.53 | 0.599 |  | 0.14 | 55 | 0.00 | 1.000 |
|  | rs1993116 | C | 0.67 | 97 | 0.84 | 0.401 |  | 0.67 | 85 | -0.35 | 0.728 |
|  |  | T | 0.33 | 97 | -0.84 | 0.401 |  | 0.33 | 85 | 0.35 | 0.728 |
|  | rs10500804 | A | 0.56 | 101 | -0.98 | 0.326 |  | 0.56 | 89 | 0.36 | 0.720 |
|  |  | C | 0.44 | 101 | 0.98 | 0.326 |  | 0.44 | 89 | -0.36 | 0.720 |
|  | rs1562902 | C | 0.48 | 100 | -0.81 | 0.415 |  | 0.48 | 86 | 0.95 | 0.345 |
|  |  | T | 0.52 | 100 | 0.81 | 0.415 |  | 0.52 | 86 | -0.95 | 0.345 |
| CYP27B1 | rs1048691 | A | 0.27 | 76 | 0.49 | 0.627 |  | 0.27 | 63 | 1.11 | 0.267 |
|  |  | G | 0.73 | 76 | -0.49 | 0.627 |  | 0.73 | 63 | -1.11 | 0.267 |
|  | rs4646536 | C | 0.33 | 86 | 0.00 | 1.000 |  | 0.33 | 74 | -0.52 | 0.604 |
|  |  | T | 0.67 | 86 | 0.00 | 1.000 |  | 0.67 | 74 | 0.52 | 0.604 |
|  | rs8176341 | C | 0.75 | 49 | 1.55 | 0.120 |  | 0.75 | 46 | 1.21 | 0.225 |
|  |  | G | 0.25 | 49 | -1.55 | 0.120 |  | 0.25 | 46 | -1.21 | 0.225 |
| SKIIP | rs176965 | A | 0.37 | 82 | -0.02 | 0.981 |  | 0.37 | 71 | 0.14 | 0.892 |
|  |  | C | 0.63 | 82 | 0.02 | 0.981 |  | 0.63 | 71 | -0.14 | 0.892 |
|  | rs1030151 | A | 0.19 | 56 | 0.11 | 0.913 |  | 0.19 | 51 | -1.17 | 0.242 |
|  |  | G | 0.81 | 56 | -0.11 | 0.913 |  | 0.81 | 51 | 1.17 | 0.242 |
|  | rs4346144 | A | 0.13 | 55 | 0.98 | 0.329 |  | 0.13 | 47 | -0.11 | 0.915 |
|  |  | G | 0.87 | 55 | -0.98 | 0.329 |  | 0.87 | 47 | 0.11 | 0.915 |
|  | rs1477261 | A | 0.17 | 71 | -1.35 | 0.176 |  | 0.17 | 63 | -1.23 | 0.218 |
|  |  | T | 0.83 | 71 | 1.35 | 0.176 |  | 0.83 | 63 | 1.23 | 0.218 |
|  | rs11621593 | C | 0.85 | 68 | -1.01 | 0.312 |  | 0.85 | 59 | 0.19 | 0.847 |
|  |  | T | 0.15 | 68 | 1.01 | 0.312 |  | 0.15 | 59 | -0.19 | 0.847 |
|  | rs2277917 | C | 0.53 | 118 | 0.88 | 0.381 |  | 0.53 | 106 | -0.21 | 0.835 |
|  |  | G | 0.47 | 118 | -0.88 | 0.381 |  | 0.47 | 106 | 0.21 | 0.835 |
|  | rs11628795 | A | 0.21 | 95 | 1.22 | 0.224 |  | 0.21 | 78 | 0.55 | 0.581 |
|  |  | C | 0.79 | 95 | -1.22 | 0.224 |  | 0.79 | 78 | -0.55 | 0.581 |
| CYP24A1 | rs8124792 | C | 0.95 | 22 | 2.18 | **0.030** |  | 0.95 | 24 | 1.89 | 0.058 |
|  |  | T | 0.05 | 22 | -2.18 | **0.030** |  | 0.05 | 24 | -1.89 | 0.058 |
|  | rs6097801 | C | 0.83 | 84 | 1.05 | 0.293 |  | 0.83 | 65 | 1.44 | 0.149 |
|  |  | T | 0.17 | 84 | -1.05 | 0.293 |  | 0.17 | 65 | -1.44 | 0.149 |
|  | rs927650 | C | 0.55 | 108 | -1.65 | 0.100 |  | 0.55 | 91 | -1.95 | 0.051 |
|  |  | T | 0.45 | 108 | 1.65 | 0.100 |  | 0.45 | 91 | 1.95 | 0.051 |
|  | rs912505 | C | 0.29 | 89 | -1.63 | 0.104 |  | 0.29 | 80 | -2.44 | **0.015** |
|  |  | T | 0.71 | 89 | 1.63 | 0.104 |  | 0.71 | 80 | 2.44 | **0.015** |
|  | rs6068816 | C | 0.86 | 71 | -0.97 | 0.331 |  | 0.86 | 59 | 0.09 | 0.926 |
|  |  | T | 0.14 | 71 | 0.97 | 0.331 |  | 0.14 | 59 | -0.09 | 0.926 |
|  | rs2248359 | C | 0.58 | 112 | 2.15 | **0.032** |  | 0.58 | 97 | 0.56 | 0.577 |
|  |  | T | 0.42 | 112 | -2.15 | **0.032** |  | 0.42 | 97 | -0.56 | 0.577 |
|  | rs2426498 | C | 0.92 | 53 | 1.13 | 0.260 |  | 0.92 | 46 | 1.26 | 0.208 |
|  |  | T | 0.08 | 53 | -1.13 | 0.260 |  | 0.08 | 46 | -1.26 | 0.208 |

*Number of informative families to conduct the test.

P values below 0.05 are shown in bold.
