# Supplementary material for: Asthma and genes encoding components of the vitamin D pathway
Source: Respir Res. 2009 Oct 24;10(1):98. doi: 10.1186/1465-9921-10-98 (PMC2779188; doi:10.1186/1465-9921-10-98)
Supplement: Additional file 7 — Single SNP association results for asthma and atopy in the replication samples. Table showing the results for all SNPs in the replication samples. [file 1465-9921-10-98-S7.DOC]

**Supplementary Table 3**. Single SNP association results for asthma and atopy in the replication samples.

|  |  |  | **Asthma** | | | | | | | | | | | | | |  | **Atopy** | | | | | | | | | | | | | |
| --- | --- | --- | --- | --- | --- | --- | --- | --- | --- | --- | --- | --- | --- | --- | --- | --- | --- | --- | --- | --- | --- | --- | --- | --- | --- | --- | --- | --- | --- | --- | --- |
|  |  |  | **SAGE** | | | |  | **CAPPS** | | | |  | **BHS** | | | |  | **SAGE** | | | |  | **CAPPS** | | | |  | **BHS** | | | |
| **Genes** | **SNPs** | **Allele** | **AF** | **Fa** | **Z** | **p** |  | **AF** | **Fa** | **Z** | **p** |  | **AF**  **case** | **AF**  **ctrl** | **X^2^** | **p** |  | **AF** | **Fa** | **Z** | **p** |  | **AF** | **Fa** | **Z** | **p** |  | **AF**  **case** | **AF**  **ctrl** | **X^2^** | **p** |
| IL10 | rs4844553 | A | 0.07 | 25 | 0.96 | 0.336 |  | 0.05 | 11 | 0.3 | 0.763 |  | 0.05 | 0.06 | 1.21 | 0.272 |  | 0.07 | 35 | -0.16 | 0.873 |  | 0.05 | 22 | -1.88 | 0.061 |  | 0.05 | 0.06 | 0.12 | 0.729 |
|  |  | G | 0.93 | 25 | -0.96 | 0.336 |  | 0.95 | 11 | -0.3 | 0.763 |  |  |  |  |  |  | 0.93 | 35 | 0.16 | 0.873 |  | 0.95 | 22 | 1.88 | 0.061 |  |  |  |  |  |
|  | rs3024505 | A | 0.15 | 66 | 1.27 | 0.204 |  | 0.15 | 21 | -0.6 | 0.549 |  | 0.16 | 0.16 | 0.01 | 0.933 |  | 0.15 | 74 | 1.09 | 0.275 |  | 0.15 | 48 | 0.54 | 0.586 |  | 0.16 | 0.16 | 0.08 | 0.772 |
|  |  | G | 0.86 | 66 | -1.27 | 0.204 |  | 0.85 | 21 | 0.6 | 0.549 |  |  |  |  |  |  | 0.86 | 74 | -1.09 | 0.275 |  | 0.85 | 48 | -0.54 | 0.586 |  |  |  |  |  |
|  | rs3024498 | A | 0.78 | 83 | 3.06 | **0.002** |  | 0.78 | 40 | -2.56 | **0.01** |  | 0.7 | 0.72 | 1.74 | 0.187 |  | 0.78 | 108 | 1.22 | 0.223 |  | 0.78 | 70 | -0.54 | 0.592 |  | 0.71 | 0.72 | 0.26 | 0.608 |
|  |  | G | 0.23 | 83 | -3.06 | **0.002** |  | 0.22 | 40 | 2.56 | **0.01** |  |  |  |  |  |  | 0.23 | 108 | -1.22 | 0.223 |  | 0.22 | 70 | 0.54 | 0.592 |  |  |  |  |  |
|  | rs3024509 | A | 0.94 | 26 | -1.13 | 0.257 |  | 0.95 | 11 | -0.3 | 0.763 |  | 0.96 | 0.94 | 3.42 | 0.064 |  | 0.94 | 35 | 0.16 | 0.873 |  | 0.95 | 22 | 1.88 | 0.061 |  | 0.95 | 0.94 | 1.15 | 0.284 |
|  |  | G | 0.06 | 26 | 1.13 | 0.257 |  | 0.06 | 11 | 0.3 | 0.763 |  |  |  |  |  |  | 0.06 | 35 | -0.16 | 0.873 |  | 0.06 | 22 | -1.88 | 0.061 |  |  |  |  |  |
|  | rs3024492 | A | 0.78 | 81 | 2.89 | **0.004** |  | 0.78 | 40 | -2.56 | **0.01** |  | 0.7 | 0.72 | 1.55 | 0.213 |  | 0.78 | 108 | 1.13 | 0.26 |  | 0.78 | 69 | -0.75 | 0.453 |  | 0.71 | 0.72 | 0.20 | 0.655 |
|  |  | T | 0.22 | 81 | -2.89 | **0.004** |  | 0.22 | 40 | 2.56 | **0.01** |  |  |  |  |  |  | 0.22 | 108 | -1.13 | 0.26 |  | 0.22 | 69 | 0.75 | 0.453 |  |  |  |  |  |
|  | rs3024490 | A | 0.28 | 69 | 0.63 | 0.527 |  | 0.29 | 34 | -1.64 | 0.101 |  | 0.24 | 0.22 | 1.63 | 0.202 |  | 0.28 | 99 | 0.36 | 0.717 |  | 0.29 | 78 | 0 | 1 |  | 0.23 | 0.23 | 0.07 | 0.788 |
|  |  | C | 0.72 | 69 | -0.63 | 0.527 |  | 0.71 | 34 | 1.64 | 0.101 |  |  |  |  |  |  | 0.72 | 99 | -0.36 | 0.717 |  | 0.71 | 78 | 0 | 1 |  |  |  |  |  |
|  | rs1800872 | A | 0.28 | 70 | 0.73 | 0.463 |  | 0.3 | 34 | -1.64 | 0.101 |  | 0.24 | 0.22 | 1.64 | 0.2 |  | 0.28 | 98 | 0.46 | 0.649 |  | 0.3 | 78 | -0.1 | 0.922 |  | 0.23 | 0.23 | 0.07 | 0.786 |
|  |  | C | 0.72 | 70 | -0.73 | 0.463 |  | 0.7 | 34 | 1.64 | 0.101 |  |  |  |  |  |  | 0.72 | 98 | -0.46 | 0.649 |  | 0.7 | 78 | 0.1 | 0.922 |  |  |  |  |  |
|  | rs1800896 | C | 0.43 | 87 | -1.22 | 0.221 |  | 0.43 | 40 | 2.29 | **0.022** |  | 0.5 | 0.5 | 0.02 | 0.889 |  | 0.43 | 124 | -0.55 | 0.583 |  | 0.43 | 83 | 0.29 | 0.772 |  | 0.50 | 0.50 | 0.06 | 0.804 |
|  |  | T | 0.57 | 87 | 1.22 | 0.221 |  | 0.58 | 40 | -2.29 | **0.022** |  |  |  |  |  |  | 0.57 | 124 | 0.55 | 0.583 |  | 0.58 | 83 | -0.29 | 0.772 |  |  |  |  |  |
|  | rs10494879 | C | 0.63 | 93 | 1.44 | 0.151 |  | 0.64 | 42 | -2.07 | **0.039** |  | 0.55 | 0.56 | 0.51 | 0.476 |  | 0.63 | 127 | 0.08 | 0.938 |  | 0.64 | 82 | -1.18 | 0.239 |  | 0.55 | 0.56 | 0.13 | 0.717 |
|  |  | G | 0.37 | 93 | -1.44 | 0.151 |  | 0.36 | 42 | 2.07 | **0.039** |  |  |  |  |  |  | 0.37 | 127 | -0.08 | 0.938 |  | 0.36 | 82 | 1.18 | 0.239 |  |  |  |  |  |
| IL1RL1 | rs4090473 | C | 0.49 | 98 | 0 | 1 |  | 0.52 | 40 | -0.4 | 0.691 |  | 0.51 | 0.51 | 0.06 | 0.811 |  | 0.49 | 139 | -0.88 | 0.381 |  | 0.52 | 86 | -1.11 | 0.269 |  | 0.51 | 0.51 | 0.14 | 0.709 |
|  |  | G | 0.51 | 98 | 0 | 1 |  | 0.48 | 40 | 0.4 | 0.691 |  |  |  |  |  |  | 0.51 | 139 | 0.88 | 0.381 |  | 0.48 | 86 | 1.11 | 0.269 |  |  |  |  |  |
|  | rs950880 | A | 0.37 | 91 | 0.29 | 0.774 |  | 0.38 | 44 | -0.38 | 0.701 |  | 0.4 | 0.39 | 0 | 0.95 |  | 0.37 | 128 | 0.46 | 0.643 |  | 0.38 | 94 | -0.45 | 0.652 |  | 0.41 | 0.37 | 2.78 | 0.096 |
|  |  | C | 0.63 | 91 | -0.29 | 0.774 |  | 0.62 | 44 | 0.38 | 0.701 |  |  |  |  |  |  | 0.63 | 128 | -0.46 | 0.643 |  | 0.62 | 94 | 0.45 | 0.652 |  |  |  |  |  |
|  | rs1420089 | C | 0.09 | 39 | 1.41 | 0.16 |  | 0.09 | 19 | -0.23 | 0.819 |  | 0.11 | 0.11 | 0.08 | 0.782 |  | 0.09 | 50 | 1.91 | 0.057 |  | 0.09 | 40 | 0 | 1 |  | 0.12 | 0.12 | 0.03 | 0.874 |
|  |  | T | 0.91 | 39 | -1.41 | 0.16 |  | 0.91 | 19 | 0.23 | 0.819 |  |  |  |  |  |  | 0.91 | 50 | -1.91 | 0.057 |  | 0.91 | 40 | 0 | 1 |  |  |  |  |  |
|  | rs1420103 | A | 0.31 | 82 | -0.69 | 0.49 |  | 0.28 | 33 | 0.93 | 0.355 |  | 0.24 | 0.24 | 0.05 | 0.819 |  | 0.31 | 111 | -0.25 | 0.802 |  | 0.28 | 82 | 1.41 | 0.157 |  | 0.23 | 0.24 | 0.04 | 0.848 |
|  |  | C | 0.69 | 82 | 0.69 | 0.49 |  | 0.72 | 33 | -0.93 | 0.355 |  |  |  |  |  |  | 0.69 | 111 | 0.25 | 0.802 |  | 0.72 | 82 | -1.41 | 0.157 |  |  |  |  |  |
|  | rs1041973 | A | 0.22 | 80 | -0.1 | 0.921 |  | 0.23 | 35 | 0.16 | 0.876 |  |  |  |  |  |  | 0.22 | 98 | -1.99 | **0.046** |  | 0.23 | 73 | 0.11 | 0.915 |  |  |  |  |  |
|  |  | C | 0.78 | 80 | 0.1 | 0.921 |  | 0.77 | 35 | -0.16 | 0.876 |  |  |  |  |  |  | 0.78 | 98 | 1.99 | **0.046** |  | 0.77 | 73 | -0.11 | 0.915 |  |  |  |  |  |
|  | rs6719130 | C | 0.89 | 47 | 0.26 | 0.793 |  | 0.89 | 25 | 0.38 | 0.705 |  | 0.85 | 0.86 | 0.57 | 0.451 |  | 0.89 | 63 | 0.11 | 0.909 |  | 0.89 | 43 | 0.3 | 0.768 |  | 0.85 | 0.85 | 0.01 | 0.931 |
|  |  | T | 0.11 | 47 | -0.26 | 0.793 |  | 0.11 | 25 | -0.38 | 0.705 |  |  |  |  |  |  | 0.11 | 63 | -0.11 | 0.909 |  | 0.11 | 43 | -0.3 | 0.768 |  |  |  |  |  |
|  | rs3771175 | A | 0.13 | 57 | -0.36 | 0.722 |  | 0.14 | 28 | -0.18 | 0.857 |  | 0.11 | 0.12 | 0.14 | 0.709 |  | 0.13 | 68 | -1.77 | 0.077 |  | 0.14 | 50 | -0.93 | 0.354 |  | 0.11 | 0.13 | 2.30 | 0.130 |
|  |  | T | 0.87 | 57 | 0.36 | 0.722 |  | 0.86 | 28 | 0.18 | 0.857 |  |  |  |  |  |  | 0.87 | 68 | 1.77 | 0.077 |  | 0.86 | 50 | 0.93 | 0.354 |  |  |  |  |  |
|  | rs1946131 | A | 0.11 | 43 | -0.3 | 0.768 |  | 0.1 | 16 | -0.23 | 0.819 |  | 0.1 | 0.1 | 0.36 | 0.55 |  | 0.11 | 52 | -1.05 | 0.294 |  | 0.1 | 37 | 0.78 | 0.435 |  | 0.10 | 0.09 | 1.61 | 0.205 |
|  |  | G | 0.9 | 43 | 0.3 | 0.768 |  | 0.9 | 16 | 0.23 | 0.819 |  |  |  |  |  |  | 0.9 | 52 | 1.05 | 0.294 |  | 0.9 | 37 | -0.78 | 0.435 |  |  |  |  |  |
|  | rs1921622 | C | 0.52 | 95 | 0.09 | 0.928 |  | 0.49 | 41 | 0.9 | 0.37 |  | 0.44 | 0.46 | 0.68 | 0.411 |  | 0.52 | 133 | -0.53 | 0.595 |  | 0.49 | 94 | 0.98 | 0.325 |  | 0.44 | 0.48 | 2.83 | 0.092 |
|  |  | T | 0.48 | 95 | -0.09 | 0.928 |  | 0.51 | 41 | -0.9 | 0.37 |  |  |  |  |  |  | 0.48 | 133 | 0.53 | 0.595 |  | 0.51 | 94 | -0.98 | 0.325 |  |  |  |  |  |
|  | rs10204837 | A | 0.33 | 88 | 0.48 | 0.629 |  | 0.35 | 43 | -0.4 | 0.691 |  | 0.37 | 0.37 | 0.02 | 0.884 |  | 0.33 | 115 | 0 | 1 |  | 0.35 | 91 | -0.83 | 0.409 |  | 0.37 | 0.40 | 2.45 | 0.118 |
|  |  | C | 0.67 | 88 | -0.48 | 0.629 |  | 0.65 | 43 | 0.4 | 0.691 |  |  |  |  |  |  | 0.67 | 115 | 0 | 1 |  | 0.65 | 91 | 0.83 | 0.409 |  |  |  |  |  |
|  | rs11465567 | A | 0.9 | 42 | 0.45 | 0.655 |  | 0.9 | 16 | 0.23 | 0.819 |  | 0.9 | 0.9 | 0.25 | 0.614 |  | 0.9 | 52 | 1.31 | 0.189 |  | 0.9 | 38 | -0.46 | 0.647 |  | 0.90 | 0.91 | 1.58 | 0.209 |
|  |  | G | 0.11 | 42 | -0.45 | 0.655 |  | 0.11 | 16 | -0.23 | 0.819 |  |  |  |  |  |  | 0.11 | 52 | -1.31 | 0.189 |  | 0.11 | 38 | 0.46 | 0.647 |  |  |  |  |  |
| CD86 | rs12106790 | G | 0.19 | 69 | -1.24 | 0.216 |  | 0.19 | 32 | 1.44 | 0.15 |  | 0.19 | 0.2 | 1.41 | 0.235 |  | 0.19 | 89 | -0.99 | 0.322 |  | 0.19 | 67 | 0.11 | 0.909 |  | 0.20 | 0.20 | 0.04 | 0.851 |
|  |  | T | 0.81 | 69 | 1.24 | 0.216 |  | 0.81 | 32 | -1.44 | 0.15 |  |  |  |  |  |  | 0.81 | 89 | 0.99 | 0.322 |  | 0.81 | 67 | -0.11 | 0.909 |  |  |  |  |  |
|  | rs2681404 | C | 0.11 | 36 | 0.8 | 0.423 |  | 0.11 | 22 | 0.39 | 0.695 |  | 0.15 | 0.14 | 0.4 | 0.526 |  | 0.11 | 56 | -0.39 | 0.696 |  | 0.11 | 32 | 1.03 | 0.303 |  | 0.15 | 0.13 | 2.66 | 0.103 |
|  |  | T | 0.89 | 36 | -0.8 | 0.423 |  | 0.89 | 22 | -0.39 | 0.695 |  |  |  |  |  |  | 0.89 | 56 | 0.39 | 0.696 |  | 0.89 | 32 | -1.03 | 0.303 |  |  |  |  |  |
|  | rs2715275 | C | 0.17 | 46 | 0.94 | 0.345 |  | 0.17 | 28 | 1.06 | 0.289 |  | 0.2 | 0.2 | 0.1 | 0.749 |  | 0.17 | 78 | 0.1 | 0.917 |  | 0.17 | 52 | 0.14 | 0.893 |  | 0.21 | 0.20 | 0.55 | 0.458 |
|  |  | T | 0.83 | 46 | -0.94 | 0.345 |  | 0.84 | 28 | -1.06 | 0.289 |  |  |  |  |  |  | 0.83 | 78 | -0.1 | 0.917 |  | 0.84 | 52 | -0.14 | 0.893 |  |  |  |  |  |
|  | rs2681408 | C | 0.87 | 45 | -0.14 | 0.886 |  | 0.85 | 20 | -0.66 | 0.513 |  | 0.88 | 0.89 | 1.13 | 0.288 |  | 0.87 | 66 | 0.23 | 0.816 |  | 0.85 | 57 | 0.49 | 0.622 |  | 0.89 | 0.88 | 1.53 | 0.216 |
|  |  | T | 0.13 | 45 | 0.14 | 0.886 |  | 0.15 | 20 | 0.66 | 0.513 |  |  |  |  |  |  | 0.13 | 66 | -0.23 | 0.816 |  | 0.15 | 57 | -0.49 | 0.622 |  |  |  |  |  |
|  | rs4308217 | A | 0.36 | 98 | -0.35 | 0.724 |  | 0.32 | 37 | -1.86 | 0.063 |  | 0.32 | 0.32 | 0 | 0.992 |  | 0.36 | 122 | 0.24 | 0.808 |  | 0.32 | 86 | -0.09 | 0.925 |  | 0.32 | 0.32 | 0.00 | 0.946 |
|  |  | C | 0.64 | 98 | 0.35 | 0.724 |  | 0.69 | 37 | 1.86 | 0.063 |  |  |  |  |  |  | 0.64 | 122 | -0.24 | 0.808 |  | 0.69 | 86 | 0.09 | 0.925 |  |  |  |  |  |
|  | rs9282641 | C | 0.93 | 36 | 0.8 | 0.423 |  | 0.93 | 14 | -0.78 | 0.439 |  | 0.89 | 0.92 | 7.34 | **0.007** |  | 0.93 | 42 | 0 | 1 |  | 0.93 | 35 | -0.16 | 0.873 |  | 0.91 | 0.90 | 0.82 | 0.365 |
|  |  | T | 0.07 | 36 | -0.8 | 0.423 |  | 0.07 | 14 | 0.78 | 0.439 |  |  |  |  |  |  | 0.07 | 42 | 0 | 1 |  | 0.07 | 35 | 0.16 | 0.873 |  |  |  |  |  |
|  | rs9831894 | G | 0.42 | 88 | -1.12 | 0.261 |  | 0.4 | 40 | -2.61 | **0.009** |  | 0.39 | 0.39 | 0 | 0.952 |  | 0.42 | 124 | -0.23 | 0.817 |  | 0.4 | 96 | -0.26 | 0.792 |  | 0.39 | 0.40 | 0.21 | 0.645 |
|  |  | T | 0.58 | 88 | 1.12 | 0.261 |  | 0.6 | 40 | 2.61 | **0.009** |  |  |  |  |  |  | 0.58 | 124 | 0.23 | 0.817 |  | 0.6 | 96 | 0.26 | 0.792 |  |  |  |  |  |
|  | rs11717893 | C | 0.23 | 69 | 0.33 | 0.739 |  | 0.22 | 37 | 1.51 | 0.132 |  | 0.26 | 0.26 | 0.22 | 0.642 |  | 0.23 | 97 | -0.47 | 0.641 |  | 0.22 | 66 | 1.81 | 0.07 |  | 0.27 | 0.26 | 0.46 | 0.498 |
|  |  | T | 0.78 | 69 | -0.33 | 0.739 |  | 0.78 | 37 | -1.51 | 0.132 |  |  |  |  |  |  | 0.78 | 97 | 0.47 | 0.641 |  | 0.78 | 66 | -1.81 | 0.07 |  |  |  |  |  |
|  | rs2681415 | A | 0.86 | 53 | -0.64 | 0.522 |  | 0.82 | 24 | -0.58 | 0.564 |  | 0.85 | 0.87 | 1.99 | 0.159 |  | 0.86 | 74 | -0.87 | 0.383 |  | 0.82 | 62 | -0.23 | 0.816 |  | 0.86 | 0.86 | 0.01 | 0.930 |
|  |  | G | 0.14 | 53 | 0.64 | 0.522 |  | 0.18 | 24 | 0.58 | 0.564 |  |  |  |  |  |  | 0.14 | 74 | 0.87 | 0.383 |  | 0.18 | 62 | 0.23 | 0.816 |  |  |  |  |  |
|  | rs3792285 | G | 0.89 | 45 | -0.14 | 0.886 |  | 0.9 | 22 | -1.63 | 0.102 |  | 0.89 | 0.88 | 0.04 | 0.838 |  | 0.89 | 61 | 0 | 1 |  | 0.9 | 44 | -1.18 | 0.238 |  | 0.89 | 0.87 | 1.18 | 0.278 |
|  |  | T | 0.11 | 45 | 0.14 | 0.886 |  | 0.1 | 22 | 1.63 | 0.102 |  |  |  |  |  |  | 0.11 | 61 | 0 | 1 |  | 0.1 | 44 | 1.18 | 0.238 |  |  |  |  |  |
|  | rs2332096 | G | 0.57 | 87 | -1.53 | 0.127 |  | 0.56 | 43 | -2.36 | **0.018** |  | 0.53 | 0.55 | 0.47 | 0.491 |  | 0.57 | 129 | -1.08 | 0.28 |  | 0.56 | 94 | -0.92 | 0.357 |  | 0.54 | 0.54 | 0.00 | 0.958 |
|  |  | T | 0.44 | 87 | 1.53 | 0.127 |  | 0.44 | 43 | 2.36 | **0.018** |  |  |  |  |  |  | 0.44 | 129 | 1.08 | 0.28 |  | 0.44 | 94 | 0.92 | 0.357 |  |  |  |  |  |
|  | rs1915092 | A | 0.51 | 90 | 1.45 | 0.147 |  | 0.56 | 44 | 1.6 | 0.109 |  | 0.58 | 0.57 | 0 | 0.968 |  | 0.51 | 128 | 1.13 | 0.26 |  | 0.56 | 96 | 0.73 | 0.465 |  | 0.58 | 0.58 | 0.03 | 0.866 |
|  |  | T | 0.49 | 90 | -1.45 | 0.147 |  | 0.44 | 44 | -1.6 | 0.109 |  |  |  |  |  |  | 0.49 | 128 | -1.13 | 0.26 |  | 0.44 | 96 | -0.73 | 0.465 |  |  |  |  |  |
|  | rs9848900 | A | 0.74 | 86 | -0.85 | 0.393 |  | 0.7 | 35 | -0.48 | 0.631 |  | 0.73 | 0.73 | 0 | 0.968 |  | 0.74 | 113 | -1.59 | 0.112 |  | 0.7 | 75 | 0.84 | 0.399 |  | 0.73 | 0.73 | 0.16 | 0.688 |
|  |  | G | 0.27 | 86 | 0.85 | 0.393 |  | 0.31 | 35 | 0.48 | 0.631 |  |  |  |  |  |  | 0.27 | 113 | 1.59 | 0.112 |  | 0.31 | 75 | -0.84 | 0.399 |  |  |  |  |  |
|  | rs10804556 | C | 0.2 | 71 | -0.43 | 0.666 |  | 0.24 | 29 | 0 | 1 |  | 0.21 | 0.2 | 0.28 | 0.598 |  | 0.2 | 99 | 0.55 | 0.581 |  | 0.24 | 68 | -0.22 | 0.823 |  | 0.20 | 0.20 | 0.03 | 0.868 |
|  |  | T | 0.8 | 71 | 0.43 | 0.666 |  | 0.76 | 29 | 0 | 1 |  |  |  |  |  |  | 0.8 | 99 | -0.55 | 0.581 |  | 0.76 | 68 | 0.22 | 0.823 |  |  |  |  |  |
|  | rs1129055 | C | 0.74 | 83 | 0.39 | 0.695 |  | 0.7 | 30 | -0.32 | 0.746 |  | 0.71 | 0.71 | 0.14 | 0.71 |  | 0.74 | 112 | -0.08 | 0.933 |  | 0.7 | 72 | -0.53 | 0.596 |  | 0.71 | 0.71 | 0.01 | 0.934 |
|  |  | T | 0.26 | 83 | -0.39 | 0.695 |  | 0.31 | 30 | 0.32 | 0.746 |  |  |  |  |  |  | 0.26 | 112 | 0.08 | 0.933 |  | 0.31 | 72 | 0.53 | 0.596 |  |  |  |  |  |
|  | rs1915087 | C | 0.31 | 81 | 0.58 | 0.56 |  | 0.36 | 34 | -0.15 | 0.879 |  | 0.34 | 0.33 | 0.04 | 0.84 |  | 0.31 | 117 | 1.25 | 0.213 |  | 0.36 | 77 | -0.4 | 0.686 |  | 0.34 | 0.34 | 0.02 | 0.879 |
|  |  | T | 0.7 | 81 | -0.58 | 0.56 |  | 0.64 | 34 | 0.15 | 0.879 |  |  |  |  |  |  | 0.7 | 117 | -1.25 | 0.213 |  | 0.64 | 77 | 0.4 | 0.686 |  |  |  |  |  |
|  | rs2681401 | A | 0.48 | 87 | 0 | 1 |  | 0.5 | 39 | -1.82 | 0.069 |  | 0.41 | 0.4 | 0.02 | 0.886 |  | 0.48 | 124 | 0.48 | 0.633 |  | 0.5 | 85 | -0.75 | 0.454 |  | 0.41 | 0.40 | 0.10 | 0.749 |
|  |  | C | 0.52 | 87 | 0 | 1 |  | 0.5 | 39 | 1.82 | 0.069 |  |  |  |  |  |  | 0.52 | 124 | -0.48 | 0.633 |  | 0.5 | 85 | 0.75 | 0.454 |  |  |  |  |  |
|  | rs6765945 | G | 0.3 | 91 | 1.12 | 0.261 |  | 0.32 | 32 | 1.72 | 0.086 |  | 0.27 | 0.3 | 3.07 | 0.08 |  | 0.3 | 113 | 0.75 | 0.455 |  | 0.32 | 78 | 0.2 | 0.84 |  | 0.28 | 0.29 | 0.22 | 0.636 |
|  |  | T | 0.7 | 91 | -1.12 | 0.261 |  | 0.68 | 32 | -1.72 | 0.086 |  |  |  |  |  |  | 0.7 | 113 | -0.75 | 0.455 |  | 0.68 | 78 | -0.2 | 0.84 |  |  |  |  |  |
| CYP2R1 | rs1868997 | A | 0.37 | 81 | 0.29 | 0.77 |  | 0.37 | 31 | 0.16 | 0.876 |  | 0.38 | 0.36 | 2.46 | 0.117 |  | 0.37 | 120 | -0.16 | 0.873 |  | 0.37 | 84 | -1.3 | 0.194 |  | 0.36 | 0.37 | 0.02 | 0.881 |
|  |  | G | 0.63 | 81 | -0.29 | 0.77 |  | 0.63 | 31 | -0.16 | 0.876 |  |  |  |  |  |  | 0.63 | 120 | 0.16 | 0.873 |  | 0.63 | 84 | 1.3 | 0.194 |  |  |  |  |  |
|  | rs11023371 | C | 0.92 | 31 | 0 | 1 |  | 0.93 | 16 | 0.47 | 0.637 |  | 0.91 | 0.93 | 3.6 | 0.058 |  | 0.92 | 44 | 0.56 | 0.579 |  | 0.93 | 36 | 0.65 | 0.516 |  | 0.93 | 0.92 | 0.00 | 0.951 |
|  |  | T | 0.08 | 31 | 0 | 1 |  | 0.07 | 16 | -0.47 | 0.637 |  |  |  |  |  |  | 0.08 | 44 | -0.56 | 0.579 |  | 0.07 | 36 | -0.65 | 0.516 |  |  |  |  |  |
|  | rs11023374 | C | 0.27 | 72 | -0.63 | 0.532 |  | 0.27 | 35 | 0.44 | 0.662 |  | 0.27 | 0.28 | 0.07 | 0.793 |  | 0.27 | 91 | 0.38 | 0.703 |  | 0.27 | 72 | 1.05 | 0.292 |  | 0.28 | 0.27 | 0.08 | 0.775 |
|  |  | T | 0.73 | 72 | 0.63 | 0.532 |  | 0.73 | 35 | -0.44 | 0.662 |  |  |  |  |  |  | 0.73 | 91 | -0.38 | 0.703 |  | 0.73 | 72 | -1.05 | 0.292 |  |  |  |  |  |
|  | rs10500804 | G | 0.4 | 92 | 1.81 | 0.07 |  | 0.44 | 40 | -0.41 | 0.68 |  | 0.41 | 0.44 | 4.25 | **0.039** |  | 0.4 | 119 | 0.72 | 0.47 |  | 0.44 | 94 | 0.81 | 0.421 |  | 0.42 | 0.43 | 0.30 | 0.582 |
|  |  | T | 0.6 | 92 | -1.81 | 0.07 |  | 0.56 | 40 | 0.41 | 0.68 |  |  |  |  |  |  | 0.6 | 119 | -0.72 | 0.47 |  | 0.56 | 94 | -0.81 | 0.421 |  |  |  |  |  |
|  | rs1562902 | C | 0.46 | 93 | -0.83 | 0.405 |  | 0.42 | 39 | -0.42 | 0.674 |  | 0.47 | 0.43 | 4.71 | **0.03** |  | 0.46 | 126 | 0.16 | 0.874 |  | 0.42 | 93 | -0.62 | 0.538 |  | 0.46 | 0.43 | 2.38 | 0.123 |
|  |  | T | 0.54 | 93 | 0.83 | 0.405 |  | 0.58 | 39 | 0.42 | 0.674 |  |  |  |  |  |  | 0.54 | 126 | -0.16 | 0.874 |  | 0.58 | 93 | 0.62 | 0.538 |  |  |  |  |  |
| CYP24A1 | rs6097797 | A | 0.83 | 60 | -0.12 | 0.906 |  | 0.84 | 24 | -0.37 | 0.715 |  | 0.85 | 0.86 | 1.54 | 0.215 |  | 0.83 | 79 | -0.42 | 0.673 |  | 0.84 | 49 | -1.66 | 0.096 |  | 0.86 | 0.85 | 0.68 | 0.409 |
|  |  | G | 0.17 | 60 | 0.12 | 0.906 |  | 0.16 | 24 | 0.37 | 0.715 |  |  |  |  |  |  | 0.17 | 79 | 0.42 | 0.673 |  | 0.16 | 49 | 1.66 | 0.096 |  |  |  |  |  |
|  | rs8124792 | A | 0.06 | 30 | 0.17 | 0.862 |  | 0.08 | 12 | -0.26 | 0.796 |  | 0.06 | 0.06 | 0.29 | 0.591 |  | 0.06 | 42 | 1.18 | 0.238 |  | 0.08 | 40 | 0.15 | 0.881 |  | 0.07 | 0.05 | 4.65 | **0.031** |
|  |  | G | 0.94 | 30 | -0.17 | 0.862 |  | 0.93 | 12 | 0.26 | 0.796 |  |  |  |  |  |  | 0.94 | 42 | -1.18 | 0.238 |  | 0.93 | 40 | -0.15 | 0.881 |  |  |  |  |  |
|  | rs927650 | C | 0.54 | 94 | 0.81 | 0.417 |  | 0.57 | 41 | -0.69 | 0.492 |  | 0.55 | 0.5 | 6.75 | **0.009** |  | 0.54 | 127 | 0.23 | 0.816 |  | 0.57 | 100 | 0.78 | 0.439 |  | 0.54 | 0.52 | 0.89 | 0.344 |
|  |  | T | 0.46 | 94 | -0.81 | 0.417 |  | 0.43 | 41 | 0.69 | 0.492 |  |  |  |  |  |  | 0.46 | 127 | -0.23 | 0.816 |  | 0.43 | 100 | -0.78 | 0.439 |  |  |  |  |  |
|  | rs912505 | A | 0.75 | 75 | 0.53 | 0.596 |  | 0.73 | 29 | 0.69 | 0.493 |  | 0.78 | 0.79 | 0.41 | 0.521 |  | 0.75 | 106 | 0.83 | 0.409 |  | 0.73 | 86 | -0.2 | 0.843 |  | 0.77 | 0.79 | 1.64 | 0.200 |
|  |  | G | 0.25 | 75 | -0.53 | 0.596 |  | 0.27 | 29 | -0.69 | 0.493 |  |  |  |  |  |  | 0.25 | 106 | -0.83 | 0.409 |  | 0.27 | 86 | 0.2 | 0.843 |  |  |  |  |  |
|  | rs6068816 | C | 0.9 | 39 | 0.31 | 0.758 |  | 0.89 | 12 | -0.54 | 0.593 |  | 0.9 | 0.89 | 0.02 | 0.875 |  | 0.9 | 65 | 0.7 | 0.485 |  | 0.89 | 40 | -0.3 | 0.768 |  | 0.89 | 0.89 | 0.14 | 0.708 |
|  |  | T | 0.1 | 39 | -0.31 | 0.758 |  | 0.11 | 12 | 0.54 | 0.593 |  |  |  |  |  |  | 0.1 | 65 | -0.7 | 0.485 |  | 0.11 | 40 | 0.3 | 0.768 |  |  |  |  |  |
|  | rs4809960 | A | 0.75 | 75 | 0.42 | 0.677 |  | 0.74 | 33 | 0 | 1 |  | 0.76 | 0.76 | 0.01 | 0.916 |  | 0.75 | 110 | 0.09 | 0.93 |  | 0.74 | 76 | -1.01 | 0.312 |  | 0.74 | 0.77 | 2.44 | 0.118 |
|  |  | G | 0.25 | 75 | -0.42 | 0.677 |  | 0.26 | 33 | 0 | 1 |  |  |  |  |  |  | 0.25 | 110 | -0.09 | 0.93 |  | 0.26 | 76 | 1.01 | 0.312 |  |  |  |  |  |
|  | rs2248359 | A | 0.35 | 87 | 1.04 | 0.296 |  | 0.44 | 41 | 1.82 | 0.069 |  | 0.41 | 0.41 | 0.02 | 0.889 |  | 0.35 | 133 | 0.54 | 0.588 |  | 0.44 | 97 | 2.51 | **0.012** |  | 0.42 | 0.40 | 1.14 | 0.287 |
|  |  | G | 0.65 | 87 | -1.04 | 0.296 |  | 0.56 | 41 | -1.82 | 0.069 |  |  |  |  |  |  | 0.65 | 133 | -0.54 | 0.588 |  | 0.56 | 97 | -2.51 | **0.012** |  |  |  |  |  |
|  | rs2426498 | C | 0.87 | 49 | -1.39 | 0.166 |  | 0.87 | 24 | 0.82 | 0.414 |  | 0.86 | 0.87 | 0.03 | 0.859 |  | 0.87 | 58 | -2.14 | **0.032** |  | 0.87 | 61 | 0.25 | 0.806 |  | 0.86 | 0.87 | 0.06 | 0.814 |
|  |  | G | 0.13 | 49 | 1.39 | 0.166 |  | 0.13 | 24 | -0.82 | 0.414 |  |  |  |  |  |  | 0.13 | 58 | 2.14 | **0.032** |  | 0.13 | 61 | -0.25 | 0.806 |  |  |  |  |  |
|  | rs6068821 | A | 0.41 | 95 | 2.32 | **0.021** |  | 0.45 | 39 | 0.27 | 0.785 |  | 0.41 | 0.39 | 1.15 | 0.283 |  | 0.41 | 126 | 2.24 | **0.025** |  | 0.45 | 96 | 1.17 | 0.241 |  | 0.42 | 0.40 | 1.07 | 0.302 |
|  |  | G | 0.59 | 95 | -2.32 | **0.021** |  | 0.55 | 39 | -0.27 | 0.785 |  |  |  |  |  |  | 0.59 | 126 | -2.24 | **0.025** |  | 0.55 | 96 | -1.17 | 0.241 |  |  |  |  |  |

CAPPS, Canadian Asthma Primary Prevention Study; SAGE, Study of Asthma Genes and the Environment; BHS, Busselton Health Study. AF, allele frequency; Fa, Number of informative families to conduct the test.

P values below 0.05 are shown in bold.
