# Supplementary material for: Asthma and genes encoding components of the vitamin D pathway
Source: Respir Res. 2009 Oct 24;10(1):98. doi: 10.1186/1465-9921-10-98 (PMC2779188; doi:10.1186/1465-9921-10-98)
Supplement: Additional file 9 — Single SNP association results for asthma and atopy in combined analyses (SLSJ, CAPPS, SAGE, and BHS). Table showing the results for the combined analyses. [file 1465-9921-10-98-S9.DOC]

**Supplementary Table 4**. Single SNP association results for asthma and atopy in combined analyses (SLSJ, CAPPS, SAGE, and BHS).

|  |  | **Asthma** | | | | |  | **Atopy** | | | | |
| --- | --- | --- | --- | --- | --- | --- | --- | --- | --- | --- | --- | --- |
| **Gene** | **rs** | **Allele** | **Allele Freq**  **Cases** | **Allele**  **Freq**  **Controls** | **χ^2^** | **P value** |  | **Allele** | **Allele Freq**  **Cases** | **Allele**  **Freq**  **Controls** | **χ^2^** | **P value** |
| CD86 | rs12106790 | T | 0.82 | 0.80 | 2.45 | 0.1177 |  | T | 0.81 | 0.81 | 0.08 | 0.7716 |
|  | rs2681404 | C | 0.14 | 0.13 | 0.72 | 0.3961 |  | C | 0.14 | 0.12 | 1.71 | 0.1916 |
|  | rs2715275 | T | 0.81 | 0.80 | 0.00 | 0.9844 |  | C | 0.20 | 0.19 | 0.48 | 0.4867 |
|  | rs2681408 | T | 0.12 | 0.11 | 1.05 | 0.3060 |  | C | 0.89 | 0.88 | 1.40 | 0.2368 |
|  | rs4308217 | C | 0.68 | 0.67 | 0.02 | 0.8895 |  | A | 0.33 | 0.32 | 0.03 | 0.8612 |
|  | rs9282641 | T | 0.11 | 0.08 | 4.80 | **0.0284** |  | C | 0.91 | 0.90 | 0.66 | 0.4176 |
|  | rs9831894 | T | 0.61 | 0.60 | 0.24 | 0.6256 |  | T | 0.61 | 0.60 | 0.27 | 0.6044 |
|  | rs11717893 | C | 0.26 | 0.25 | 0.31 | 0.5800 |  | C | 0.25 | 0.25 | 0.16 | 0.6870 |
|  | rs2681415 | G | 0.15 | 0.13 | 2.32 | 0.1278 |  | G | 0.14 | 0.14 | 0.24 | 0.6212 |
|  | rs3792285 | G | 0.89 | 0.88 | 0.02 | 0.8902 |  | G | 0.89 | 0.88 | 0.98 | 0.3225 |
|  | rs2332096 | T | 0.47 | 0.45 | 1.41 | 0.2351 |  | T | 0.46 | 0.45 | 0.30 | 0.5808 |
|  | rs1915092 | A | 0.57 | 0.56 | 0.36 | 0.5470 |  | A | 0.57 | 0.56 | 0.48 | 0.4898 |
|  | rs9848900 | G | 0.28 | 0.27 | 0.10 | 0.7547 |  | G | 0.28 | 0.27 | 0.17 | 0.6794 |
|  | rs10804556 | C | 0.21 | 0.20 | 0.09 | 0.7587 |  | C | 0.21 | 0.20 | 0.02 | 0.9013 |
|  | rs1129055 | C | 0.72 | 0.71 | 0.25 | 0.6205 |  | T | 0.29 | 0.29 | 0.01 | 0.9106 |
|  | rs1915087 | C | 0.33 | 0.33 | 0.17 | 0.6823 |  | C | 0.34 | 0.33 | 0.50 | 0.4780 |
|  | rs2681401 | A | 0.42 | 0.41 | 0.02 | 0.8938 |  | A | 0.42 | 0.42 | 0.26 | 0.6121 |
|  | rs6765945 | T | 0.72 | 0.70 | 1.36 | 0.2435 |  | T | 0.71 | 0.71 | 0.00 | 0.9499 |
| CYP24A1 | rs6097797 | G | 0.16 | 0.14 | 1.38 | 0.2394 |  | A | 0.85 | 0.85 | 0.27 | 0.6008 |
|  | rs8124792 | A | 0.06 | 0.06 | 0.32 | 0.5732 |  | A | 0.07 | 0.05 | 6.08 | **0.0137** |
|  | rs927650 | C | 0.55 | 0.50 | 7.63 | **0.0057** |  | C | 0.54 | 0.52 | 0.93 | 0.3345 |
|  | rs912505 | G | 0.22 | 0.22 | 0.14 | 0.7069 |  | G | 0.23 | 0.22 | 0.54 | 0.4620 |
|  | rs6068816 | C | 0.90 | 0.89 | 0.07 | 0.7934 |  | C | 0.89 | 0.89 | 0.45 | 0.5016 |
|  | rs4809960 | A | 0.76 | 0.76 | 0.07 | 0.7902 |  | G | 0.25 | 0.23 | 1.81 | 0.1782 |
|  | rs2248359 | A | 0.41 | 0.40 | 0.27 | 0.6006 |  | A | 0.41 | 0.39 | 1.43 | 0.2320 |
|  | rs2426498 | G | 0.14 | 0.13 | 0.46 | 0.4967 |  | G | 0.14 | 0.12 | 1.28 | 0.2587 |
|  | rs6068821 | A | 0.42 | 0.39 | 3.66 | 0.0558 |  | A | 0.42 | 0.39 | 3.92 | **0.0476** |
| CYP2R1 | rs1868997 | A | 0.38 | 0.35 | 2.55 | 0.1105 |  | G | 0.63 | 0.63 | 0.04 | 0.8336 |
|  | rs11023371 | T | 0.08 | 0.07 | 2.97 | 0.0849 |  | C | 0.93 | 0.92 | 0.10 | 0.7464 |
|  | rs11023374 | T | 0.73 | 0.72 | 0.23 | 0.6290 |  | C | 0.27 | 0.27 | 0.18 | 0.6693 |
|  | rs10500804 | T | 0.58 | 0.56 | 1.43 | 0.2315 |  | T | 0.58 | 0.58 | 0.03 | 0.8702 |
|  | rs1562902 | C | 0.46 | 0.44 | 2.90 | 0.0884 |  | C | 0.46 | 0.44 | 2.10 | 0.1471 |
| IL10 | rs4844553 | G | 0.95 | 0.94 | 0.39 | 0.5313 |  | G | 0.95 | 0.94 | 0.14 | 0.7051 |
|  | rs3024505 | A | 0.16 | 0.16 | 0.20 | 0.6553 |  | A | 0.16 | 0.15 | 0.06 | 0.8137 |
|  | rs3024498 | A | 0.72 | 0.72 | 0.00 | 0.9697 |  | A | 0.72 | 0.72 | 0.01 | 0.9213 |
|  | rs3024509 | A | 0.95 | 0.94 | 1.50 | 0.2203 |  | A | 0.95 | 0.94 | 1.01 | 0.3147 |
|  | rs3024492 | A | 0.72 | 0.72 | 0.00 | 0.9625 |  | A | 0.73 | 0.73 | 0.01 | 0.9058 |
|  | rs3024490 | A | 0.24 | 0.22 | 2.03 | 0.1540 |  | C | 0.77 | 0.76 | 0.00 | 0.9478 |
|  | rs1800872 | A | 0.24 | 0.22 | 2.16 | 0.1418 |  | C | 0.77 | 0.76 | 0.00 | 0.9809 |
|  | rs1800896 | T | 0.51 | 0.50 | 0.11 | 0.7431 |  | T | 0.52 | 0.51 | 0.23 | 0.6333 |
|  | rs10494879 | G | 0.44 | 0.44 | 0.01 | 0.9129 |  | G | 0.43 | 0.42 | 0.09 | 0.7692 |
| IL1RL1 | rs4090473 | G | 0.49 | 0.49 | 0.05 | 0.8238 |  | G | 0.49 | 0.49 | 0.01 | 0.9190 |
|  | rs950880 | A | 0.39 | 0.39 | 0.03 | 0.8678 |  | A | 0.40 | 0.37 | 2.78 | 0.0952 |
|  | rs1420089 | C | 0.11 | 0.10 | 0.06 | 0.8002 |  | C | 0.11 | 0.11 | 0.44 | 0.5049 |
|  | rs1420103 | C | 0.76 | 0.75 | 0.25 | 0.6191 |  | C | 0.75 | 0.75 | 0.09 | 0.7679 |
|  | rs6719130 | T | 0.15 | 0.14 | 0.35 | 0.5568 |  | C | 0.86 | 0.86 | 0.02 | 0.8980 |
|  | rs3771175 | T | 0.88 | 0.88 | 0.24 | 0.6226 |  | T | 0.89 | 0.87 | 4.81 | **0.0284** |
|  | rs1946131 | A | 0.10 | 0.10 | 0.18 | 0.6722 |  | A | 0.10 | 0.09 | 0.36 | 0.5460 |
|  | rs1921622 | T | 0.55 | 0.54 | 0.51 | 0.4742 |  | T | 0.55 | 0.52 | 2.91 | 0.0882 |
|  | rs10204837 | A | 0.37 | 0.37 | 0.00 | 0.9588 |  | C | 0.64 | 0.62 | 1.83 | 0.1761 |
|  | rs11465567 | G | 0.10 | 0.10 | 0.08 | 0.7799 |  | G | 0.10 | 0.09 | 0.21 | 0.6435 |
|  | rs1041973 | C | 0.76 | 0.75 | 0.01 | 0.9207 |  | C | 0.82 | 0.76 | 3.99 | **0.0458** |

P values lowered than 0.05 are shown in bold.
